# The Easter Egg Weevil (*Pachyrhynchus*) genome reveals syntenic patterns in Coleoptera across 200 million years of evolution

**DOI:** 10.1371/journal.pgen.1009745

**Published:** 2021-08-30

**Authors:** Matthew H. Van Dam, Analyn Anzano Cabras, James B. Henderson, Andrew J. Rominger, Cynthia Pérez Estrada, Arina D. Omer, Olga Dudchenko, Erez Lieberman Aiden, Athena W. Lam

**Affiliations:** 1 Entomology Department, Institute for Biodiversity Science and Sustainability, California Academy of Sciences, San Francisco, California, United States of America; 2 Center for Comparative Genomics, Institute for Biodiversity Science and Sustainability, California Academy of Science, San Francisco, California, United States of America; 3 Coleoptera Research Center, Institute for Biodiversity and Environment, University of Mindanao, Matina, Davao City, Philippines; 4 School of Biology and Ecology, University of Maine, Orono, Maine, United States of America; 5 The Center for Genome Architecture, Department of Molecular and Human Genetics, Baylor College of Medicine, Houston, Texas, United States of America; The University of North Carolina at Chapel Hill, UNITED STATES

## Abstract

Patterns of genomic architecture across insects remain largely undocumented or decoupled from a broader phylogenetic context. For instance, it is unknown whether translocation rates differ between insect orders. We address broad scale patterns of genome architecture across Insecta by examining synteny in a phylogenetic framework from open-source insect genomes. To accomplish this, we add a chromosome level genome to a crucial lineage, Coleoptera. Our assembly of the *Pachyrhynchus sulphureomaculatus* genome is the first chromosome scale genome for the hyperdiverse Phytophaga lineage and currently the largest insect genome assembled to this scale. The genome is significantly larger than those of other weevils, and this increase in size is caused by repetitive elements. Our results also indicate that, among beetles, there are instances of long-lasting (>200 Ma) localization of genes to a particular chromosome with few translocation events. While some chromosomes have a paucity of translocations, intra-chromosomal synteny was almost absent, with gene order thoroughly shuffled along a chromosome. This large amount of reshuffling within chromosomes with few inter-chromosomal events contrasts with patterns seen in mammals in which the chromosomes tend to exchange larger blocks of material more readily. To place our findings in an evolutionary context, we compared syntenic patterns across Insecta in a phylogenetic framework. For the first time, we find that synteny decays at an exponential rate relative to phylogenetic distance. Additionally, there are significant differences in decay rates between insect orders, this pattern was not driven by Lepidoptera alone which has a substantially different rate.

## Author summary

Patterns of genomic architecture across insects remain largely undocumented or decoupled from a broader evolutionary context. For instance, it is unknown whether rates of gene order decay differ between insect orders. We address broad scale patterns of genome architecture across Insecta by examining synteny (shared gene order) in a phylogenetic framework from open-source insect genomes (143 complete chromosome assemblies in total). To accomplish this, we add a chromosome level genome to a crucial lineage, Coleoptera (beetles). Our assembly of the Easter Egg Weevil *Pachyrhynchus sulphureomaculatus* genome is the first chromosome scale genome for the hyperdiverse Phytophaga lineage and currently the largest insect genome assembled to this scale. We are the first to identify in beetles that genes stay localized on chromosomes for hundreds of millions of years, while their order along chromosomes gets completely shuffled over time. We are also the first to empirically demonstrate that synteny decay rates different significantly between insect orders and that this pattern in not driven solely by Lepidoptera (moths and butterflies), which has a substantially different rate.

## Introduction

Beetles represent one of the most diverse groups of metazoans, with ~400,000 described species [1] and estimates of total diversity up to 0.9–2.1 million species [2]. Among beetles, weevils (Coleoptera: Curculionidae) are one of the most diverse insect groups (>60,000 described species [3]), encompassing a huge range of life history strategies and occupying every conceivable niche in a terrestrial ecosystem. With morphological forms specialized to ecological habits, such as feeding on fungi, seeds, pollen, wood, roots, and even kangaroo dung, weevils make an excellent system in which to study the evolution of different ecomorphologies [3,4]. Weevils belong to the group Phytophaga whose members comprise lineages that specialize on and have co-diversified with many plant lineages [5,6]. Given their vast diversity and economic importance as pollinators and crop pests, knowing more about the genomic architecture of beetles should be of broad applicability. However, to date, there are few available genomes resolved to chromosome level for Coleoptera and none for weevils or the hyperdiverse beetle lineage Phytophaga [7–10]. Here we present the first genome resolved to chromosome level for the Phytophaga beetle lineage *Pachyrhynchus sulphureomaculatus* Schultze, 1922 [11].

Recent advances in genome assembly techniques, such as in situ high throughput conformation capture technology (Hi-C) [12], have substantially enhanced our knowledge of genome architecture [13–15]. Increasing the accuracy and contiguity of genome assemblies has also been aided by using long-read sequencing technology in combination with in situ Hi-C [16–20]. These innovations have allowed researchers to not only reconstruct genomes to chromosome scale but also to do so relatively quickly and cheaply [21]. In addition, in situ Hi-C technology has shown that the 3D conformation of genomes is not random and that this conformation can influence gene expression and linkage [22]. The result of these new sequencing techniques has increased the number of high quality genomes for non-model insect species, including beetles [9,10,17,23–26]. Because in situ Hi-C orders scaffolds and corrects misjoins, we can study synteny (between chromosomes, unless otherwise specified) between organisms with more confidence [14,27]. In situ Hi-C is particularly important for assembling insect genomes which often have high heterozygosity as well as being composed of many repetitive elements, allowing for their assembly into chromosomes where other technologies produce significantly less contiguous and less accurate chromosome assemblies [14].

With the influx of new chromosome-level genomes, we can now begin to explore patterns of genome architecture within and between major insect lineages. For example, in Lepidoptera (butterflies and moths), genome architecture has been characterized as relatively stable with few (6%) orthologous loci being translocated [23,28–30]. Holocentric chromosomes observed throughout Lepidoptera are implicated in facilitating hybridization, [23,31–33] suggesting that genome architecture plays a significant role in their biology. In contrast to Lepidoptera, *Drosophila* species have many more translocations and rearrangements having monocentric centromeres [34]. The fungus *Cryptococcus neoformans* provides a clear example of how changing monocentric centromere position has a negative fitness costs [35]. In beetles, however, even a basic understanding of genomic architecture remains largely undocumented. The basic blueprints as revealed by in situ Hi-C maps of how a genome is organized (e.g.–with a Rabl-like conformation, i.e. grouping of telomeres and centromeres to the nuclear envelope, [36,37], holocentric chromosomes, chromosome domain territories, compartments, and topological associated domain loops) remain non-existent and therefore unplaced in a phylogenetic context. A general synthesis across insects linking these genomic architectural patterns to their function and potential influence on speciation remains incomplete. For example, do different insect orders have distinct rates of genomic rearrangements (the breakage of synteny between genes), or are the patterns we observe merely due their phylogenetic structure? The null expectation would be that there is no difference in synteny decay rate between insect orders. For the first time, we demonstrate that different insect orders do have distinct rates of synteny decay. To help accomplish this we also provide a new chromosome-level genome for Coleoptera.

## Results

### Sequencing and assembly results

Our goal was to obtain a genome with high contiguity and accuracy, we implemented a long-read sequencing strategy using PacBio long-reads in combination illumina short-reads that use in situ Hi-C library techniques to correct and reorder the scaffolds generated from the PacBio read assembly. From our PacBio library we sequenced a total of 87.5 Gbp with an N50 read length of 31,404 bp (see Table A in S1 Raw Data Reports for full report). From our in situ Hi-C library (we refer to the in situ Hi-C library or reads as “Hi-C” throughout), we sequenced a total of 228,169,567 paired reads after cleaning. Only 2.53% of our Hi-C reads were unmapped, and we had a total of 80,652,881 Hi-C contacts (intra/ inter-chromosomal interactions, i.e., chimeric read pairs). For a list of the intra-/inter-chromosomal contacts and long/short range Hi-C contacts, see Table B in S1 Raw Data Reports.

**Fig 1:**
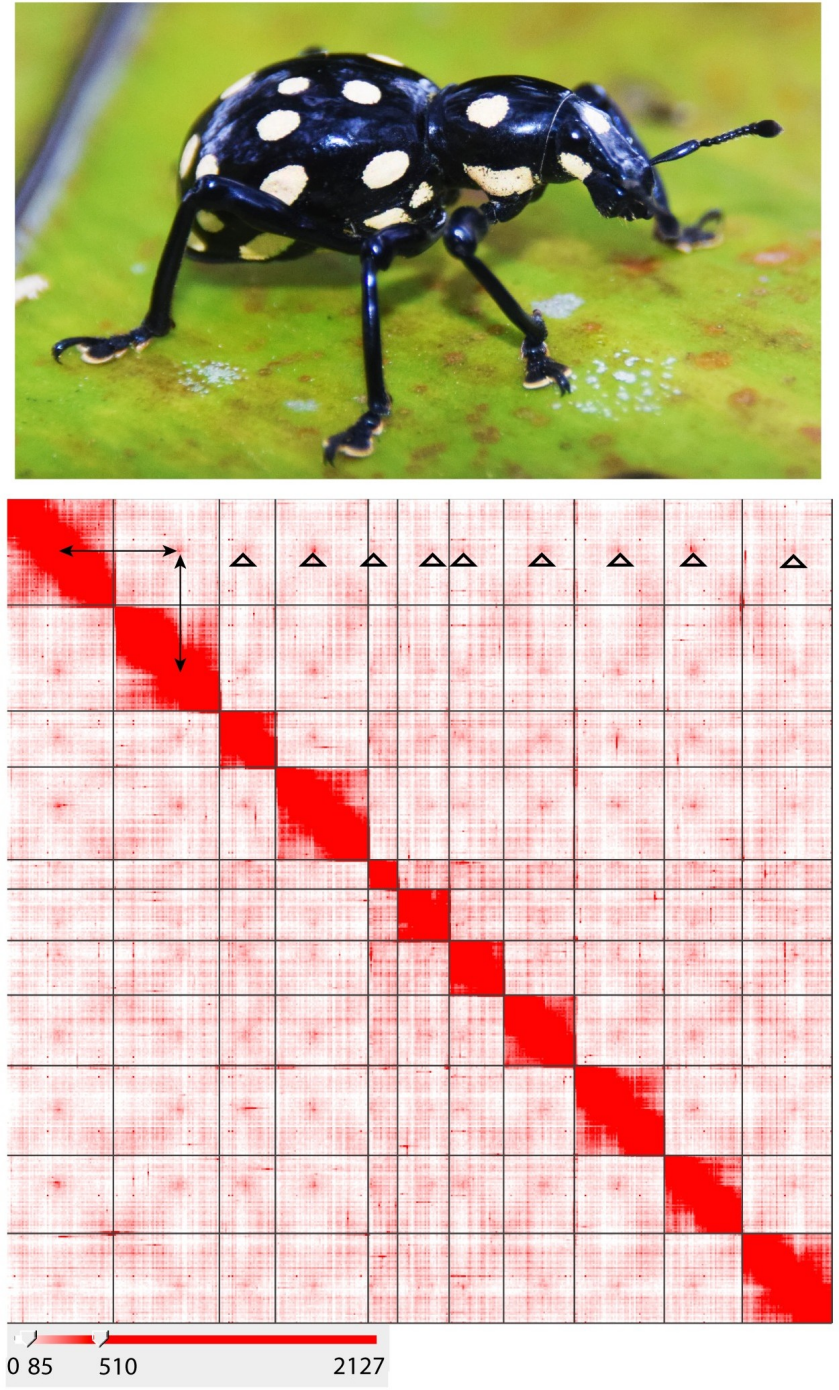
*Pachyrhynchus sulphureomaculatus*, lateral habitus. (photo by A. Cabras). Hi-C contact map heatmap of *Pachyrhynchus sulphureomaculatus* Schultze, 1922. Eleven chromosome boundaries are indicated by black lines. Heatmap scale lower left, range in counts of mapped Hi-C reads per megabase squared. Rabl-like pattern (grouping of telomeres and centromeres to the nuclear envelope) highlighted along chromosome 1, top row, top of open triangles point to contact between centromere regions, arrows indicate centromere to centromere contact between chromosomes 1 and 2. X-like pattern between adjacent off diagonal regions indicative of contact between distal portions of chromosomes.

Next to correct read errors from our initial PacBio assembly we used iterations of RACON [38] followed by collapsing duplicate haplotigs not merged in the initial assembly. Our initial PacBio assembly after 3X polishing in RACON [38] consisted of 18,240 contigs and was 2,982,578,979 bp in total length. After removing duplicate haplotigs with *Purge Haplotigs* [39], 9,751 scaffolds and 2,052,097,903 bp remained. Next, we used our Hi-C reads to order our scaffolds into chromosomes and correct misjoins. Our initial Hi-C assembly resulted in 4,111 scaffolds and 2,057,226,403 bp total. The size increase is due to 500 bp insertions of Ns (the 3D-DNA default), between scaffolds merged into super-scaffolds. Running *Pilon* (v. 1.23) in “—fix bases” mode to remove homopolymer repeats and removal of mitochondrial and contaminant scaffolds (virus or bacteria) resulted in 4,093 scaffolds and 2,051,389,195 bp in the final assembly (see Figs 1 and S1 and Tables 1 and 2). The identity of a few other scaffolds not included in the main chromosomes are ambiguous (14 potential viruses and 31 potential bacteria). We retained these but did remove any with bacteria or virus as their best blast score. From the different versions of BUSCO [40] Insecta gene sets (1658 BUSCOs version 2, 1367 version 4 and 5-beta), the percentage of complete genes varied (90.8% V2 (S2 Fig)), indicating a relatively complete assembly. Compared to other chromosome-level beetle genomes, we found a comparable number of complete BUSCO genes. However, the results vary somewhat depending on which version of BUSCO and which genes were used (S2 Fig). We found a relatively low duplication rate compared to that found in two other beetle (*Photinus* firefly [8] and *Propylea* ladybeetle [9]) genomes that used primarily long-read and Hi-C sequencing in their assembly.

**Table 1:** Summary statistics for final assembly.

Number of scaffolds	4,093
Total size of scaffolds	2,051,389,195
Number of contigs	14,365
Number of contigs in scaffolds	10,283
Number of contigs not in scaffolds	4,082
Mean scaffold size	501,195
Median scaffold size	8,175
N50 scaffold length	215,921,627
scaffold %AT	33.11
scaffold %CG	16.87
scaffold %N	0.05
**% bp of assembly in chromosomes**	**97.52**

**Table 2:** Summary statistics for final assembly by chromosome.

Chromosome	length bp	# of contigs	number of N’s (runs of 100)	percent N’s in chromo.	N50	N50 reached in # of contigs
Chr_1	263,832,947	1388	138,700	0.05%	287319	280
Chr_2	253,284,860	1222	122,100	0.04%	326447	246
Chr_3	137,890,936	683	68,200	0.04%	315991	127
Chr_4	223,502,247	1131	113,000	0.05%	297265	217
Chr_5	69,931,891	236	23,500	0.03%	452330	50
Chr_6	125,299,487	655	65,400	0.05%	304080	131
Chr_7	132,078,125	624	62,300	0.04%	368684	112
Chr_8	173,282,956	836	83,500	0.04%	335626	165
Chr_9	215,921,627	1221	122,000	0.05%	280522	237
Chr_10	186,927,849	988	98,700	0.05%	290475	197
Chr_11	218,628,933	1074	107,300	0.04%	316887	219

### Repeat content analyses

**Fig 2:**
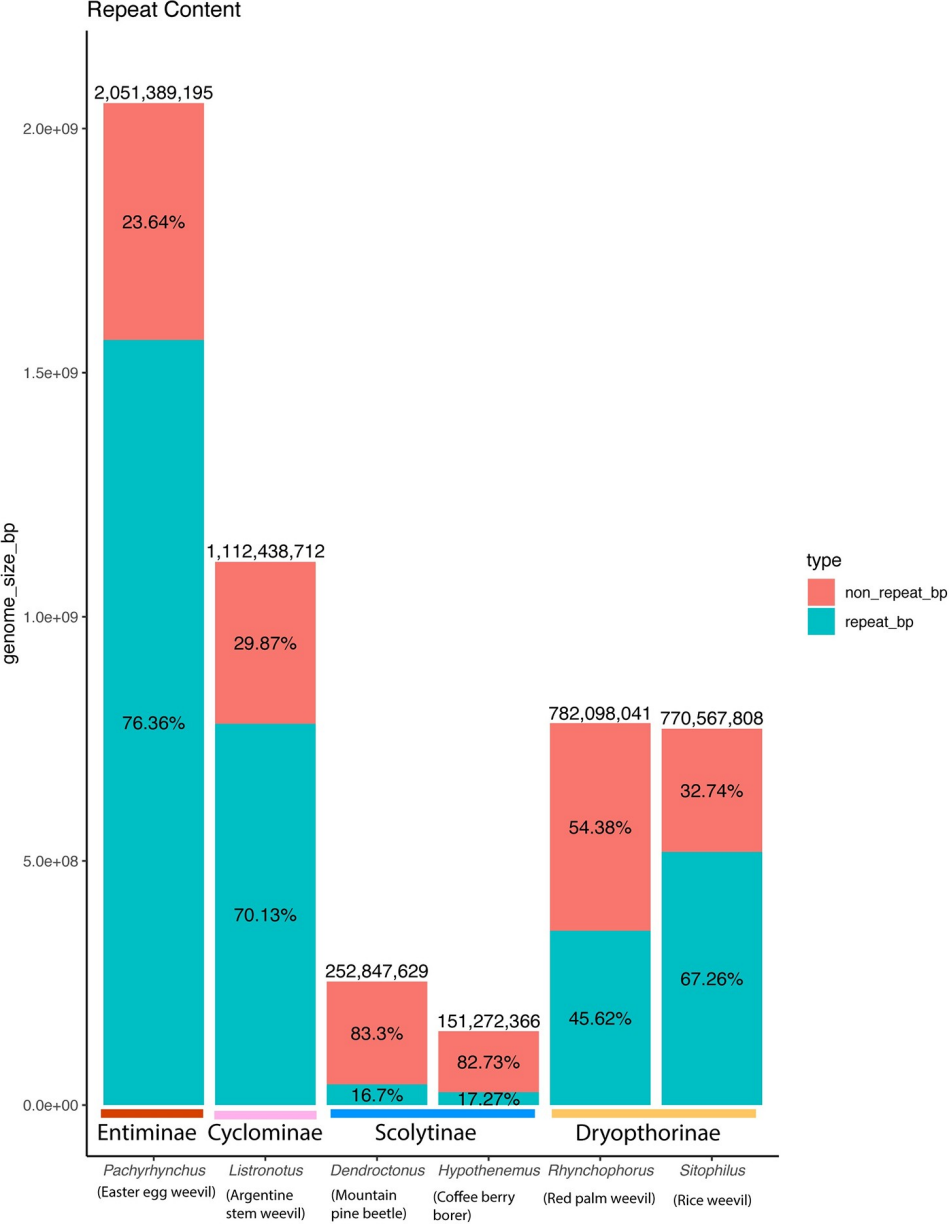
Histogram of repeat content for weevil genomes examined. Subfamily classification appears below the histograms. Latin names are in italic font with common names below in parentheses. Genome size largely corresponds to repeat content.

**Fig 3:**
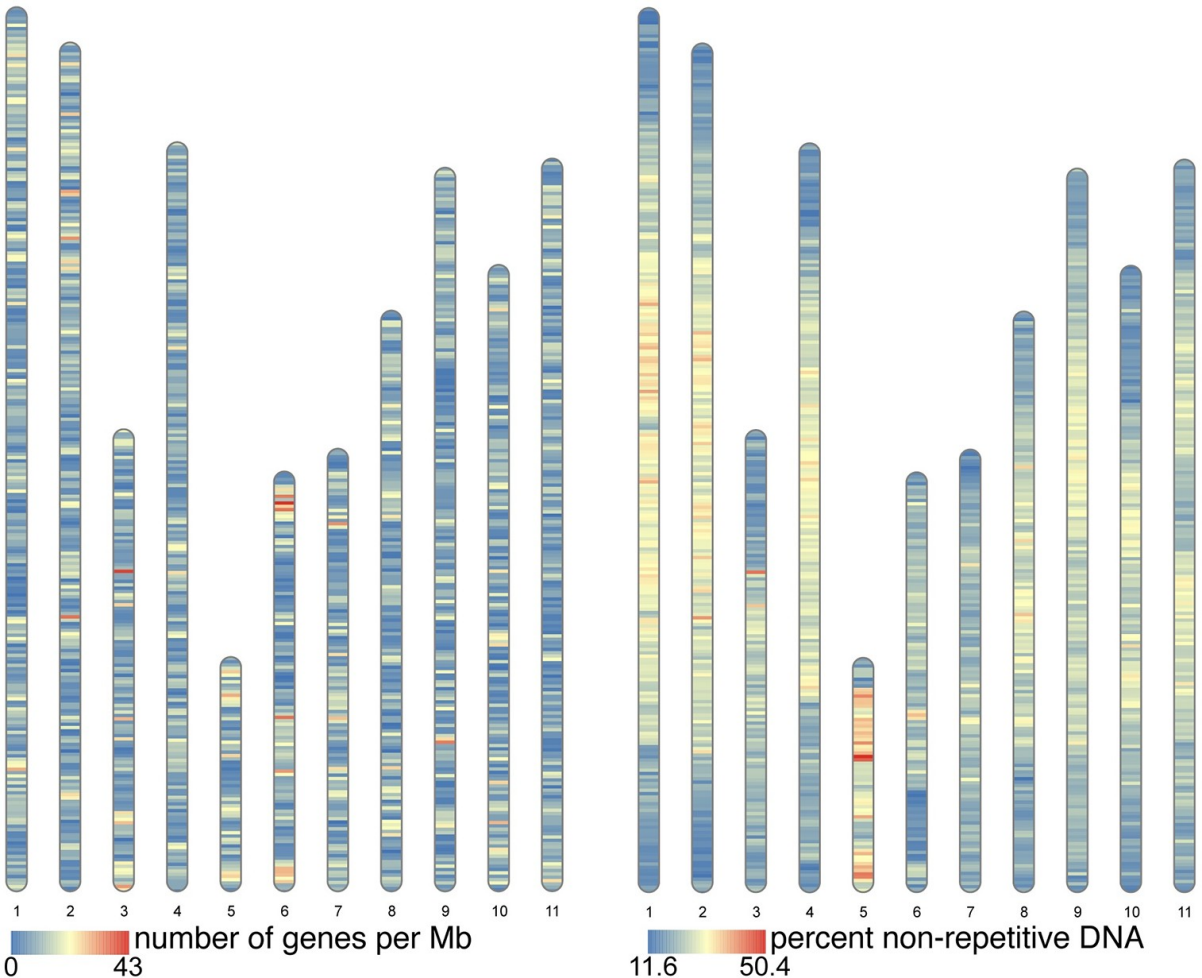
Heat map of gene density and non-repetitive DNA per 1 Mb sliding window. The 11 chromosomes are in the same order as in the Hi-C heat map (Fig 1) and fasta file of the genome. Repetitive content higher towards the distal portions of the chromosomes.

Weevils have a large distribution of genome sizes, to help investigate what is behind this pattern we analyzed each genome for its repetitive content, as repetitive content often accounts for large portions of a genome. At 2.05 Gbp, the *Pachyrhynchus sulphureomaculatus* genome is roughly 1.8 times as large as the next largest weevil (Curculionoidea) genome published to date, the 1.11 Gbp *Listronotus bonariensis*, the Argentine Stem Weevil [41], and 2.6 times the next largest, the 782 Mbp Red Palm Weevil, *Rhynchophorus ferrugineus* [42] genome. To help explain the size difference, we categorized the repeat content of *P*. *sulphureomaculatus*. The repeat content analyses from *RepeatMasker* shows that the genome of *P*. *sulphureomaculatus* consists of more than three quarters (76.36%) repetitive DNA, similar to the repeat percentage of *Listronotus*, which is the closest relative to *Pachyrhynchus*. Compared to other weevil genomes (Fig 2), *P*. *sulphureomaculatus* has roughly the same percentage of non-repetitive DNA as *Listronotus* and *Sitophilus*. However, the genomes of the two bark beetles of the subfamily Scolytinae (*Dendroctonus* and *Hypothenemus*), are ~1/12 the size of *P*. *sulphureomaculatus* and consist of only ~17% repetitive content. The *P*. *sulphureomaculatus* genome consisted of 73.1% interspersed repeats, with SINEs being 0.1%, LINEs 20.8%, LTR elements 2.6%, DNA elements 33% and unclassified repeats 16.6%. A sliding window analysis suggests that repetitive content tends to be found in a higher percentage towards the ends of the chromosomes in *P*. *sulphureomaculatus*, except in chromosome 5 (Fig 3).

### Genome annotation

As this is the first publically available weevil genome resolved to chromosome scale we wanted to provide an annotation of its genic content as this may prove an informative resource to other researchers. After removing low quality reads from our transcriptome library, a total of 20,551,938 paired reads remained. Our initial 3 transcriptome assemblies, *Trinity* de novo, *Trinity* genome guided assembly and *rnaSPAdes*, resulted in fairly similar assemblies, with each having a high number (~90%) of the BUSCO v.2 Arthropoda genes (see Table A in S1 BUSCO Analyses Results for details).

As the nuclei of cells between different species generally do not interact (except for viruses), and because Hi-C mapping will remove any non-*Pachyrhynchus* DNA from the chromosomes, we only annotated genes found within the 11 chromosomes comprising 2,000,581,858 bp. The *EVidenceModeler* analysis found that the *P*. *sulphureomaculatus* contained, 30,175 gene transcripts. After running an InterProScan (cross-referencing the results from *EVidenceModeler* with the protein databases) resulted in 18,741 gene models of which 19.01% are single exon genes. Of note are the large intron sizes on average 23,640 bp in length. For the details of results see Table 3.

**Table 3:** Results from genome annotation, lengths in bp.

Genomic class	Total	Total length	Mean length	Longest	Shortest	Mean # per gene	Mean length per gene
Genes	18,741	465,968,736	24,864	642,261	150	NA	NA
Exons	99,291–19% single exon genes	22,930,245	231	11,802	3	5	1,224
Introns	80,550	443,038,490	5,500	477,734	28	4.3	23,640

The gff, faa, gene model scores and tRNA annotations can be found Table A, Table B, Table C, and Table D in S1 Anno Results. Chromosome gene distribution is relatively even, with only a few regions enriched with genes (Fig 3). Also of note, the number of genes is larger than those found in the pine beetle *Dendroctonus ponderosae* (GCF_000355655.1: 14,342 genes) also a weevil. *P*. *sulphureomaculatus* is more similar in number to those found in other phytophagous beetles who feed on plant foliage, such as the Colorado potato beetle *Leptinotarsa decemlineata* (GCF_000500325.1: 16,533 genes) [43]. A more thorough examination of close relatives and more phylogenetically distant but ecologically similar species would need to be conducted to fully tease out why there are more gene models predicted in the foliage feeding species.

### Synteny across coleopteran chromosome-level genomes

**Fig 4:**
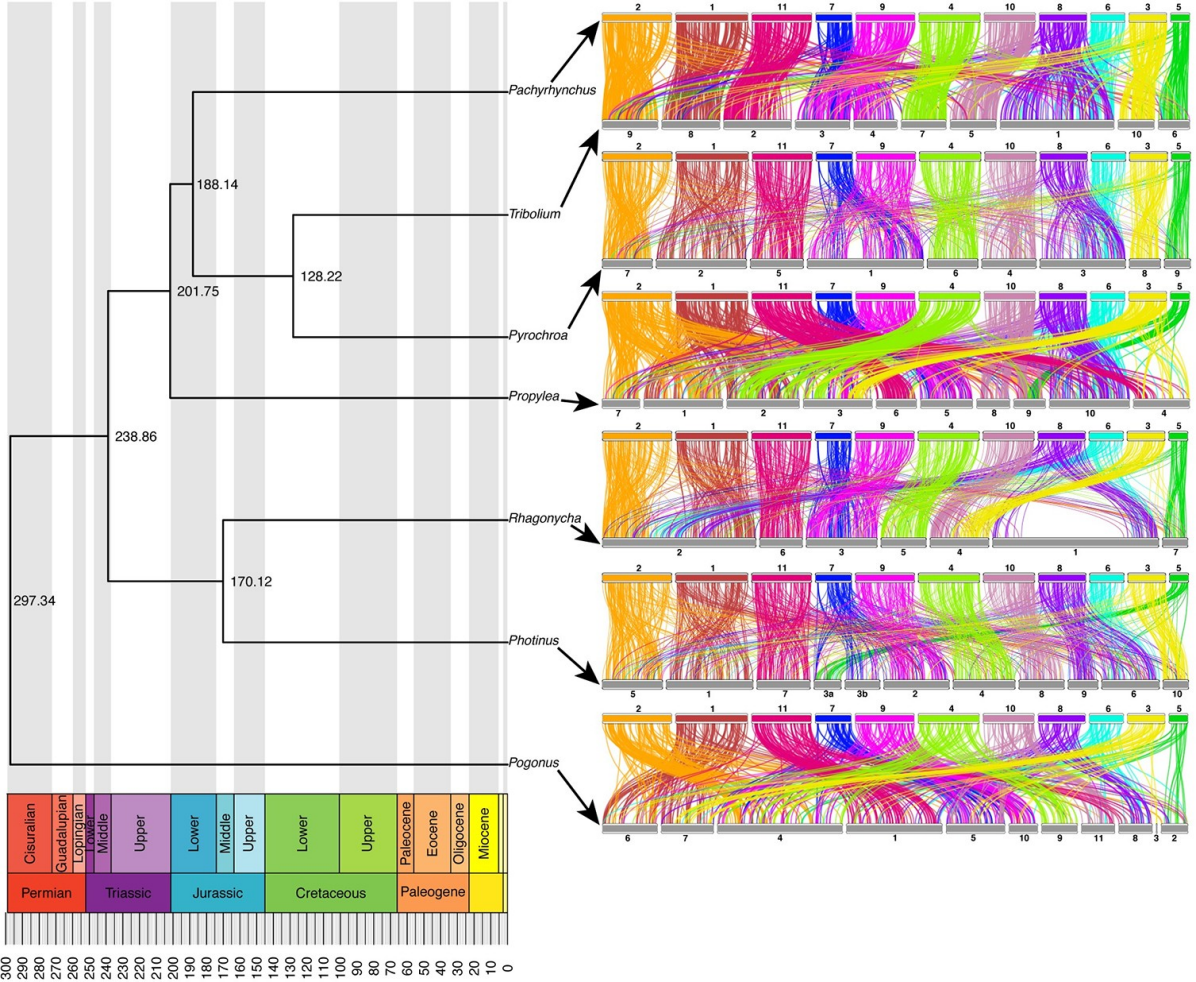
Chronogram and ideograms of 7 beetle genomes which have chromosome level assemblies. Chromosomes largely remain intact with few translocations relative to reshuffling within a chromosome. Colors correspond to the 11 chromosomes of *Pachyrhynchus sulphureomaculatus*, top row of ideogram plots. Each line represents a BUSCO gene connecting its position on the chromosome of *P*. *sulphureomaculatus* (top row, respectively) to its position on another species (lower row, respectively).

**Fig 5:**
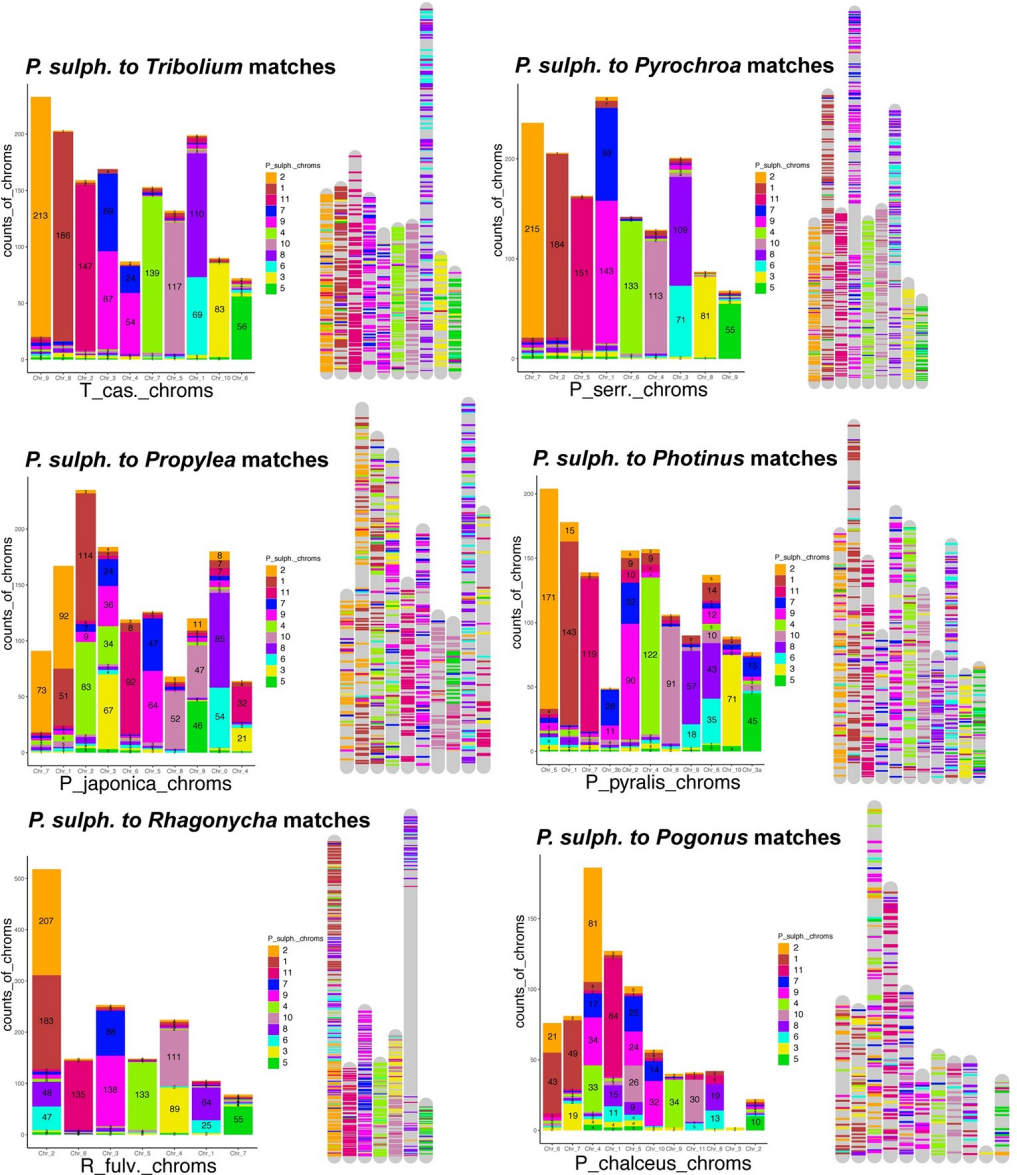
Stacked bar plots and chromosome mappings of BUSCO genes’ placements. The Y-axis represents the counts of BUSCO genes from *Pachyrhynchus sulphureomaculatus* found on the corresponding chromosomes of another species. Colors correspond to *P*. *sulphureomaculatus* chromosomes. The numbering scheme (on X-axis) of chromosomes matches the names found in the genome’s fasta file. While most chromosomes are primarily composed of one or two chromosomes, relative to *P*. *sulphureomaculatus*, the placement of the BUSCO genes are interleaved in many instances, indicating that while translocations are rare events reshuffling within a chromosome happens much more frequently.

**Fig 6:**
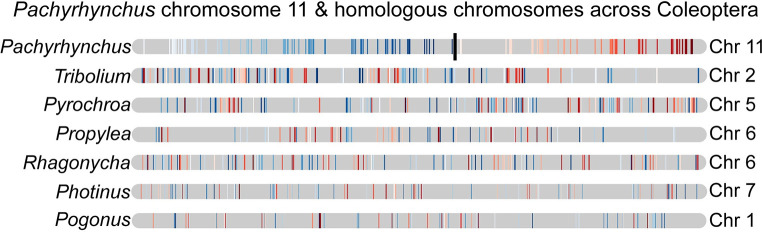
*Pachyrhynchus sulphureomaculatus* chromosome 11 and matching homologous chromosomes from taxa samples across the Coleoptera. Top row, approximate position of *Pachyrhynchus* chromosome 11 centromere marked with black line, position derived from Hi-C contact map (see Fig 1). Colored lines correspond to the position of BUSCO genes. Blue colors correspond to one chromosome arm and red colors the other. While the majority of BUSCO genes found in *Pachyrhynchus* chromosome 11 are retained in the other species there is extensive reshuffling in their positions.

Here we wanted to describe the syntenic patterns found in Coleoptera as this has not been attempted before to the best of our knowledge. To accomplish this, we mapped a BUSCO single copy gene set across the different taxa available, looking for any emergent patterns. We found that the BUSCO v.2 loci (1658 Insecta gene set), had a low level of translocations between chromosomes (Fig 4). Results show that within a chromosome, the order of BUSCO genes is not conserved (Figs 4, 5 and 6), with few long segments of synteny within a chromosome. Synteny is greatest between *P*. *sulphureomaculatus* and the five other Polyphaga beetles, and least between Adephaga (*Pogonus*) and *P*. *sulphureomaculatus*, however some of this difference may be in part to the *Pogonus* assembly having less of its contigs being localized into the chromosomes. Interestingly, there is more synteny between *P*. *sulphureomaculatus* and *Photinus pyralis* (firefly)[8] than between *P*. *sulphureomaculatus* and *Propylea japonica* (ladybird beetle), the closer relative of *P*. *sulphureomaculatus*, indicating that the lineage leading to *Propylea* has undergone many more chromosomal translocation events (Figs 4 and 5). Synteny is greatest between *P*. *sulphureomaculatus* and the two Tenebrionoidea species (*Tribolium* and *Pyrochroa*), its closest relatives.

Given the divergence time between our taxa, when translocations do occur, their initial positions are lost due to a high level of reorganization producing a pattern of interwoven segments. For example, chromosomes 8 and 9 in *Pachyrhynchus* and the large chromosome 1 in *Tribolium* (Fig 5), have no large syntenic runs of genes or obvious places of translocation. In contrast, chromosome 9 of *Propylea* and chromosome 5 of *Pachyrhynchus* are still largely intact, with the homologous segment of chromosome 5 inserted into roughly the middle of *Propylea*’s chromosome 9. Lastly, we see another 2 fusion events in the *Rhagonycha* soldier beetle. Here the chromosome number is reduced to 7 and we see 2 clear relatively recent fusion events on chromosome 2 and 4 (Fig 4). Given the relative amount of reshuffling along other parts of this chromosome, the ability to place the insertion indicates that this was a relatively recent event.

### Synteny across the insect tree of life

As we wanted to examine if insect orders have different synteny decay rates, we needed to have two pieces of information, a score for how syntenic two species are and their phylogenetic relatedness. For scoring synteny we computed the ENSEMBL Gene Order Conservations (GOC) scores [44] across all pairwise comparisons for our 143 taxa from the positions of their BUSCO version 5 genes. Species were chosen if their genome assemblies were recorded as chromosome level by NCBI or similar (using Hi-C for super scaffolding). The GOC pairwise matrix results can be found in Table A in S1 Synteny Analyses. To reconstruct the taxa’s phylogenetic relationships, we recovered 1356 BUSCO Genes in a 50% complete matrix, totaling 610,189 amino acids in length. The 50% complete matrix indicates the minimum number of taxa allowed in an alignment, loci below that percentage are removed from the analyses. The phylogenetic tree was calculated to get an estimate for the phylogenetic distance among taxa.

**Fig 7:**
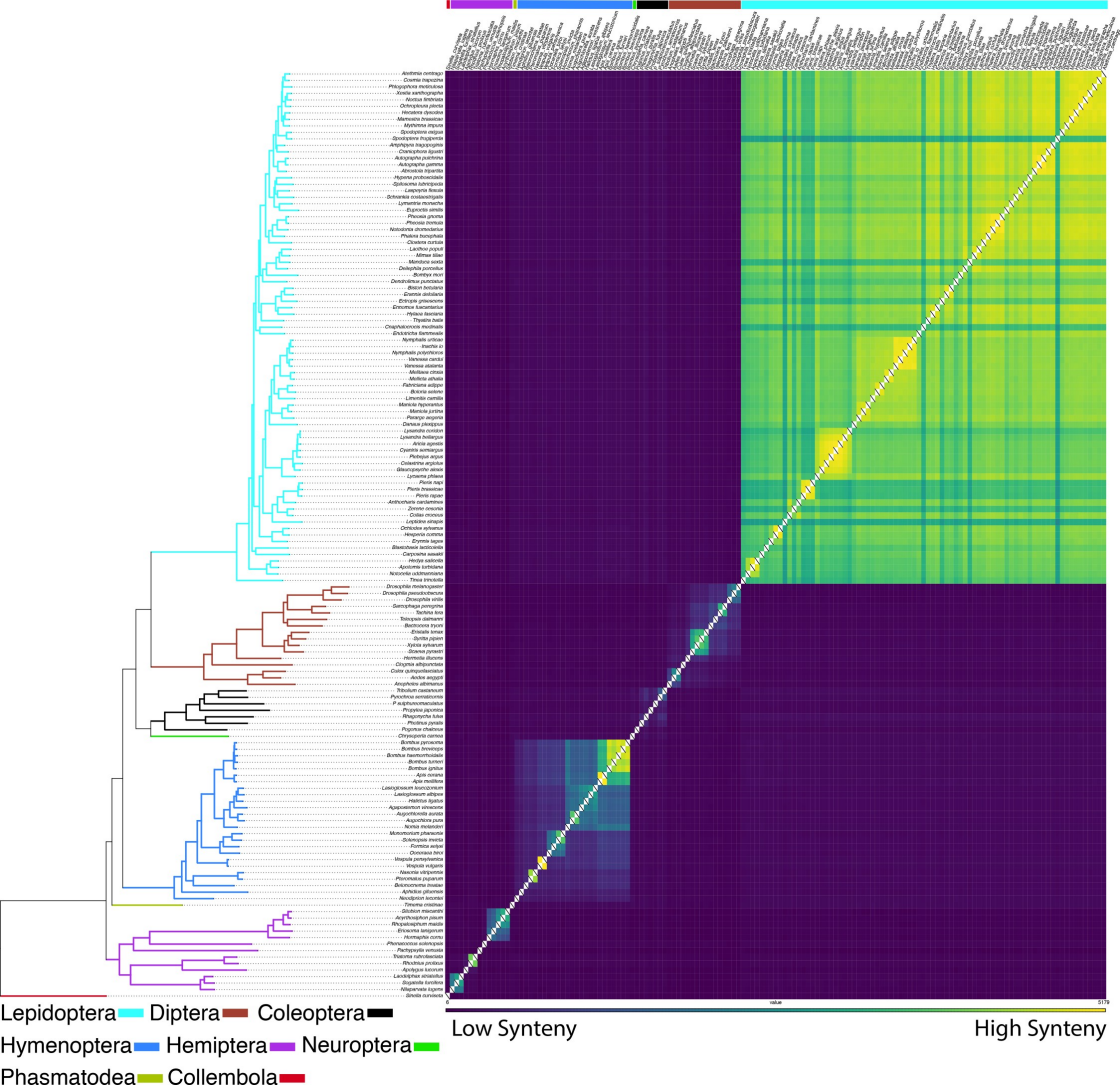
Insecta, gene order conservation score (GOC) of BUSCO genes. Left, phylogeny of taxa in analyses, derived from BUSCO genes (610,189 AA sites), reconstructed via RAxML-ng, branches colored by insect order. Right, heat map from pairwise comparisons among insects with chromosome level genomes (only genes localized to chromosomes considers in analyses). Comparisons of gene order which are more syntenic (higher GOC scores) appear in yellow boxes, dark purple indicate less synteny between taxa pairs.

**Fig 8:**
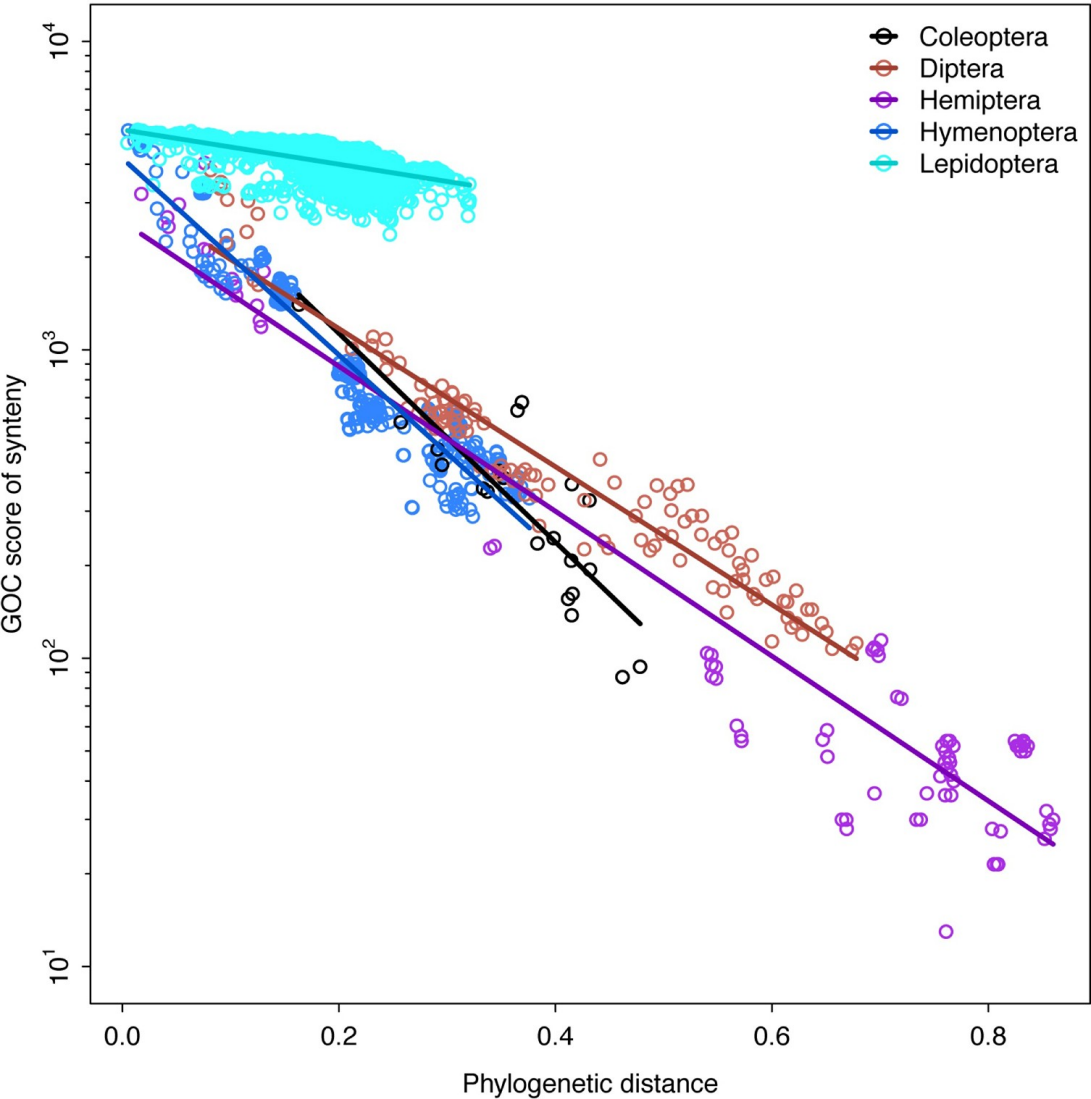
Relationship between synteny and phylogenetic distance across different insect orders. Lines show the best-fitting exponential decay model. Note the log-transformed y-axis. Phylogenetic distance is calculated from a total tree height of 1. Higher values of the GOC score indicate more synteny, lower values less synteny. Synteny decay rate of Lepidoptera differs substantially, however other insect orders also have distinct rates.

The phylogeny recovered many of the same clades as in [45] (Fig 7). While we primarily relied on chromosome scale assemblies that used Hi-C (or similar) to superscaffold into chromosomes some did not, such as the assembly of the carabid beetle *Pogonus*. Despite different assembly methods this assembly does not appear to be an outlier when we look at the synteny decay plot (Figs 7 and 8). We performed the regression analyses (below) with and without this taxon and it did not significantly alter the results, so we left it in all further analyses.

### Regression model results

As we wanted to calculate how synteny decays over phylogenetic distance and if insect orders have different rates, we first needed to avoid the lack of independence in pairwise distances (both along phylogenetic branches and in genomic position of genes) we used a permutational approach to evaluate the significance of the regression models we fit. This approach is consistent with widespread methods in ecology and evolutionary biology that preform regression analyses with distance matrices [46,47], for a full explanation see methods section.

The exponential decay model has the highest total model F-statistic and smallest p-value F9,3590 = 15,111, p = 2 × 10^−4^ (compared to linear: F9,3590 = 3,493, p = 3 × 10^−4^; power law: F9,3590 = 12,165, p = 3 × 10^−4^). This supports the exponential model as the best fitting model for the relationship between synteny and phylogenetic distance.

Using this best fitting exponential model, we then asked whether different insect orders show different rates of decay, again using permutational F-statistics. We find that the interaction between phylogenetic distance and order identity is statistically significant: F4,3590 = 1,344, p = 4 × 10^−4^. We also find that this result is not driven solely by Lepidoptera; the analysis excluding Lepidoptera still finds a significant interaction between phylogenetic distance and order: F3,511 = 39, p = 4 × 10^−4^. Results of the exponential decay model can be found in Fig 8.

## Discussion

### Hi-C and long read sequencing resolve a large complex insect genome into chromosomes

The combination of long-read DNA and Hi-C sequencing was successful in resolving a large and highly repetitive insect genome. To date, this is the largest insect genome and one of the largest arthropod genomes assembled to chromosome scale, the horseshoe crab’s (*Tachypleus tridentatus*) being only slightly larger (2.06 Gb vs 2.05 Gb) [48]. This is remarkable because the assembly of relatively large and highly repetitive insect genomes into highly contiguous ones such as this was previously unattainable [49]. Those efforts were hindered by repetitive contents breaking scaffolds or misjoining them [14,23,49]. The unusually large size of the *Pachyrhynchus* genome is mostly due to the inflated proportion of repetitive content, 76.4% of the genome (Fig 2). Again, highlighting the need for long sequencing reads to span the repetitive content. Here we used a single individual to create both our Hi-C and PacBio libraries. The main advantage over using multiple individuals is little loss of Hi-C reads mapped to the scaffolds; it also eliminates the need for isogenic lines to be established before sequencing. In our previous attempts to assemble a genome for *Pachyrhynchus*, we were greatly hindered by the loss of mappable reads when using multiple individuals. As long read sequencing improves in its capabilities of using a small amount (5–50 ng) of DNA, capitalizing on this combination of Hi-C and long-read sequencing will make it feasible to assemble chromosome scale genomes from single, very small insect specimens [19,50].

### Syntenic patterns in Coleoptera and divergent exponential decay rates of insect orders

The conserved inter-chromosomal synteny (few chromosome translocations) between the beetle genomes is surprising given the divergence times of the different lineages. For example, we recovered chromosomes that have remained 80–92% intact for more than 200 Ma (Figs 4 and 5). By contrast, the order of the BUSCO genes inside of the chromosomes are highly rearranged, such as chromosomes 8 and 6 in *Pachyrhynchus* and chromosome 1 in *Tribolium* (Figs 4 and 5). This initial finding prompted us to examine whether similar patterns are observed across other insect orders. A characteristic of Lepidoptera is having a high level of synteny across different families [23,30]. We find that relative to other insect orders sampled that Lepidoptera does have a lower rate of synteny decay. Here we performed the first formal test of this untested (but often mentioned) observation [23,30]. Previous comparisons did not take into account phylogenetic relatedness. Closely related Lepidoptera have similar levels of synteny as other similarly closely related taxa (e.g. *Bombus* and *Apis* Fig 7). But as the phylogenetic distance increase between comparisons Lepidoptera tend to have higher levels of synteny than is found in other orders. In addition to the marked difference in synteny conservation, we also found that each order has a significantly different rate of decay (Fig 8). For example, in *Drosophila*, there is less synteny between members of this genus (~40 Ma) than across all of Lepidoptera, and Coleoptera and Hymenoptera tend to decay at an even faster rate than is seen in Diptera (Fig 8). These results of gene order conservation are consistent with research of *Drosophila* topological associated domains (TADs) that showed synteny break points at approximately every 6th gene between *D*. *melanogaster*, *D*. *virilis* and *D*. *busckii*, which have a similar level of divergence as the *Drosophila* taxa we examined, about 40 Ma of divergence [34]. In addition, the chromosomal rearrangement across *Drosophila* tends to occur at TAD boundaries, not inside the loops [34,51]. In *Anopheles* mosquitos, the TAD structures seem to be associated with cytological structures as well [52]. In Diptera, despite having many breakpoints, with relatively few chromosome translocations, their chromosomes largely remain intact [53]. However, in Coleoptera, unlike Mosquitos which show each chromosomal arm being conserved [14] we do not find this same level of conservation in Coleoptera sampled. This may be due to the larger phylogenetic distance between the beetle samples. However, despite this difference we find a somewhat similar syntenic pattern between the two orders, in that the chromosomes remain intact while also being highly shuffled (Figs 4 and 5). This large amount of reshuffling within chromosomes with few inter-chromosomal events contrasts with patterns seen in mammals in which the chromosomes tend to exchange larger blocks of material more readily [54–57].

Currently, chromosome-level genomes are not available for Trichoptera (caddisflies, the sister lineage to Lepidoptera) or early diverging lineages of Lepidoptera. With the addition of these lineages, we could determine whether the observed pattern of synteny conservation is found only in Lepidopteran crown groups or whether it is more widely dispersed across the entire Lepidopteran lineage. Additionally, there are many large orders of insect without a single genome resolved to the chromosome scale or just one, e.g. Psocoptera, Thysanoptera, Neuroptera and several others. A more complete and phylogenetically even sampling of Insecta would help to aide in understanding how changes in genomic architecture may affect other processes such as speciation.

### The genomic architecture of insects and its potential impacts on speciation

Another architectural feature of *Pachyrhynchus*’ genome above the chromosome level includes the Rabl-like configuration of chromosomes, where centromeres and telomeres cluster at opposite/different regions of the nucleus. These features are important to note because they may serve an important evolutionary function, such as reducing chromosomal entanglements during interphase as well as regulating chromosomal compartmentalization [58,59]. Both major lineages of Diptera, the Nematocera (e.g. mosquitoes and Psychodidae) and Schizophora (e.g. *Drosophila*), have nucleus with a Rabl-like configuration [14,17,37]. These taxa span much of the phylogenetic distance across the dipteran lineage, and thus this pattern of chromosomal organization may be characteristic of Diptera. We also observe the Rabl-like configuration in *Pachyrhynchus* as well as in the Hi-C map of *Tribolium* (DNAZoo Consortium et al. 2020). Hi-C map observations published for the other taxa do not indicate any other obvious cases of the Rabl-like configuration within the Insecta. However, improving the quality of existing Hi-C maps would provide more evidence for this observation because a lack of valid Hi-C reads can obscure this type of chromosomal architecture.

The Hi-C maps from Tenebrionoidea and Phytophaga beetle lineages display chromosomes in the Rabl-like configuration, those of the other beetle genomes do not display this formation and are from similar tissue types to what we used [8]. It could be that this configuration is only restricted to the aforementioned lineages, more beetle genomes are required. The Rabl-like configuration is not just restricted to beetles and flies; it is also found in the yeast genome [58,60–62] as well as in wheat, barley and *Brassica* [30,63–65], and was originally described from salamander cells [36]. It is unclear how widespread the Rabl-like configuration is in Coleoptera. It is assumed that the Rabl-like configuration is found in all life stages, as appears to be the case in Diptera [14,17,52]. While the Rabl-like configuration is the predominant chromosomal arrangement observed thus far in Diptera and some Coleoptera, its evolutionary significance remains unclear. It has recently been demonstrated how changes in Condensin II impact chromosomes shape and territories which could possibly affect speciation rates by altering between few long chromosomes (with a Rabl-like configuration) and may smaller ones, as seen in Muntjac deer [66]. Our ability to detect genomic architecture’s influence on diversity, if any, is hindered by the sparse in cases, haphazard sampling of insect genomes. Rather than one to one comparison, it is more meaningful to describe patterns for a clade in a broader phylogenetic context. This will allow for the identification general patterns and potentially learning the mechanism as to why some taxa don’t fit in.

### Conclusions

In summation, we have reconstructed one of the largest and most repetitive arthropod genomes. With the combination of Hi-C reads and PacBio long-read sequencing data, we were able to resolve a highly contiguous, chromosome-level genome. Across Coleoptera, we find a novel pattern where chromosomes remain relatively intact for hundreds of millions of years with few translocation events, yet their gene order within chromosomes is completely shuffled. Lastly, we find patterns of genomic architecture are clade specific across Insecta, with different insect orders having distinct rates of synteny decay.

## Methods

### Taxon selection and natural history

*Pachyrhynchus*, from the entirely flightless tribe Pachyrhynchini, is found from the Philippines to Papua New Guinea, Australia, Taiwan, Japan, and Indonesia [11,67]. They are known for their bright, iridescent and unique elytral markings, which they use as an aposematic signal to warn predators of their unpalatability [68]. Members of other weevil groups (e.g. *Polycatus*, *Eupyrgops*, *Neopyrgops*, *Alcidodes*) and long-horned beetles (e.g. *Doliops*, *Paradoliops*) mimic *Pachyrhynchus*’ aposematic signals to ward off predators. Currently, the Pachyrynchini has 17 known genera, with the majority found exclusively in the Philippines [11,69,70].

*Pachyrhynchus* Germar, 1824 has the widest geographic range among Pachyrynchini. There are presently 145 species in the genus, of which 93% of are endemic to the Philippines [71], with the majority of species having a narrow geographic range, limited to a mountain range, island, or Pleistocene Aggregate Island Complex (PAIC) [72–74]. The general diagnostic characters of *Pachyrhynchus* Germar, 1824 include a head lacking a distinct transverse groove or distinct basal border, entire episternal suture, and antennal scape not reaching the hind eye [11]. *P*. *sulphureomaculatus* Schultze, 1922, is only recorded from Mindanao Island [11,71]. This species was described from material collected in South Cotabato but has recently been recorded (personal observations of A. Cabras) in other areas of Mindanao (e.g. Marilog, Davao City, Arakan, Cotabato, Mt. Kiamo, Bukidnon). This species belongs to the *P*. *venustus* group, conspicuous for their large size, prothorax with two dorsolateral spots in the middle a large, oblong spot at the lateral margins, and elytra with oval or oblong spots [11].

### Collection and extraction of DNA

Specimens were collected near the edge of the road in a secondary forest (HWY 81, Arakan, Cotabato, Philippines [N7.487059, E125.248795]). One individual was used for both in situ Hi-C and high molecular weight DNA libraries. A second individual was used for transcriptome sequencing. Individuals were collected live, then frozen and stored at -80°C until library preparation.

Beetle tissues were dissected carefully to avoid inclusion of contaminants from guts and impurities from chitinous cuticles. Half of the resulting tissues were used for Phenol Chloroform (PCI) based high molecular weight (HMW) DNA extraction for PacBio sequencing (the other half of the material was used as starting material for Hi-C library preparation, see below).

Tissues were homogenized on ice using a sterile razor blade. ATL buffer (140 μl) and Proteinase K (60 μl) were then added to the homogenized material and incubated at 65°C for 1 hr. The 200 μl of resulting lysate was used as starting material for the PCI extraction following a PacBio recommended protocol [75].Two additional rounds of PCI clean-up were performed to eliminate impurities such as chitin to meet the DNA requirement for PacBio sequencing. In particular, to achieve OD ratios of 1.8–2.0. DNA concentration was determined with the Qubit dsDNA HS Assay Kit (Invitrogen corp., Carlsbad, CA), and high molecular weight content was confirmed by running a Femto Pulse (Agilent, Santa Clara, USA).

### In situ Hi-C library preparation

Tissues from the same sample were homogenized using a sterile razor blade on ice. An in situ Hi-C library was prepared as described in [13] with a few modifications. Briefly, after the Streptavidin Pull-down step, the biotinylated Hi-C products underwent end repair, ligation and enrichment using the NEBNext UltraII DNA Library Preparation kit (New England Biolabs Inc, Ipswich, MA). Furthermore, titration of the number of PCR cycles was performed as described in [76].

### Transcriptome library preparation

RNA extraction was performed using tissues from a frozen sample. Tissue was extracted from the prothorax and abdomen with the digestive tract removed. The Monarch Total RNA Miniprep kit (New England Biolabs Inc, Ipswich, MA) was used for extraction. The manufacturer’s protocol for total RNA purification from tissue was followed [77]. RNA concentration was determined using the Qubit RNA HS Assay Kit (Invitrogen corp., Carlsbad, CA), and intact RNA content was confirmed by running a Bioanalyzer High Sensitivity RNA Analysis (Agilent, Santa Clara, USA). The resulting RNA was sent to Novogene Inc. for library preparation and sequencing, from which 12.5 Gbp of data were obtained.

### Genome sequencing and assembly

First, we performed an initial quality control of the in situ Hi-C library using the CPU version of *Juicer* v 1.5.7 [78] to determine if enough ligation motifs were present in the sample. To accomplish this, we first cleaned our reads with *fastp* [79] to remove sequencing adapters and low quality reads with default settings except for the more sensitive ‘—detect_adapter_for_pe’ setting on. After passing the quality control of having >30% ligation motifs present, we proceeded to sequence the full library at higher coverage. We only considered ligation motifs as this was a de novo assembly without a closely related reference genome to align to the Hi-C reads. The full Hi-C library was sequenced on a paired-end (2x150 bp) lane on an Illumina HiSeq4000. High molecular weight DNA was sent to the QB3 Genomics facility at the University of California Berkeley for sequencing on a Pacific Biosciences Sequel II platform, sequencing one cell with CLR version 2 chemistry (PacBio, Menlo Park, CA, USA).

We used *PacBio Assembly Tool Suite pb-assembly* v 0.0.8 (which includes the FALCON assembly pipeline) to assemble the primary scaffolds. Next, we polished the primary assembly using 3 rounds of mapping the raw fastq reads using *minimap2* [80] followed by using RACON [38] to help error correct the initial assembly. This was followed by running the *Purge_Haplotigs* [39] pipeline to eliminate haplotigs (alternative haplotype contigs) in the assembly. Next, using the CPU version of *Juicer* v 1.5.7, we created a site positions file for the restriction enzyme MboI using *Juicer*’s *generate_site_positions*.*py* script, followed by running *Juicer* until it creates the mapping stats file and a “*merged_nodups*” file. Then we used the 3D-DNA [14] pipeline with default settings to correct misjoins and place scaffolds into chromosome groups. After generating a Hi-C heat map, we corrected any assembly errors manually via *Juicebox Assembly Tools* v 1.11.08 [21,78]. After, (Fig 1) we ran 3D-DNA’s *run-asm-pipeline-post-review*.*sh* to produce a final assembly file and fasta. To polish our final assembly further, we aligned our Hi-C reads to our scaffolds using *bwa mem* followed by *SAMclip* and *SAMtools* ‘view’ [81] with options ‘-S -b -f 2 -q 1 -F 1536’. After grouping scaffolds into chromosomes, we divided each into a separate fasta (due to memory constraints) and used *Pilon* (v. 1.23) [82] in “—fix bases” mode as to not break our scaffolds and to fix any homopolymer repeat errors. The resulting assembly was used in all subsequent analyses.

### Removal of mitochondrial/contaminant DNA

To identify scaffolds that contained mitochondrial cytochrome oxidase subunit 1 (COI) DNA, we used BLAT v. 35 [83] using a reference sequence from *Pachyrhynchus smaragdinus* (S1 P79_coI.fasta) to query our scaffolds. Once identified, these scaffolds were removed. We also used *blast* [84] with the nt database and default settings to identify contaminant (non-arthropod or undetermined) sequences and then removed these from the final assembly. These represented only a handful of sequences.

### Repeat content analyses

To address what is making the genome of *Pachyrhynchus sulphureomaculatus* so large relative to other complete weevil genomes (>85% Benchmarking Universal Single-Copy Orthologs BUSCO Insecta genes), we compared the repeat content of *P*. *sulphureomaculatus* to 5 other weevil genomes from NCBI (see Tables A and B in S1 RepeatMasker Results). We used the de novo *RepeatModeler* v. open-1.0.11 [85] repeat set combined with all repbase recs to first model for repeat content. Next, we used *RepeatMasker* v. 4.1.0 [85] to annotate and soft mask repeat content. For *Listronotus*, we downloaded the results from [41], who used comparable methodologies. We also calculated the percentage of repetitive content (bases soft masked) in a 1 Mb sliding window across the chromosomes in *R* using a custom script.

### Genome annotation

We first cleaned our reads with *fastp* and concatenated the unpaired cleaned reads. We performed 3 different initial reconstructions of the transcriptome: 1) *Trinity* v. 2.11.0 [86,87], de novo assembly using default settings, 2) *Trinity* genome guided assembly, where we first aligned our reads with *tophat* v. 2.1.1 [88], 3) *rnaSPAdes* [89] de novo assembly. Selecting the *rnaSPAdes* assembly, because it had the most single copy *BUSCO V2* Arthropoda genes [40], we mapped our reads to this soft masked assembly using *HISAT2* v. 2.2.0 [90], and formatted a bam file using *SAMtools* ‘view -b -f 3 -F 256 -q 10’. Next, we used *BRAKER v*. *2*.*1*.*5* [91] to create an annotated gff. This process used the bam file from *HISAT2* and results from a *BUSC*O search as ‘seeding’ genes to make the resulting gff. In addition, we used the *PASA* pipeline [92,93] which used our *rnaSPAdes* transcripts aligned to the genome assembly with *BLAT* [83] and *gmap* [94]. Lastly, we used *EVidenceModeler* [93] to evaluate our different annotations using the developers’ recommended weights for each assembly type. To produce the final gene model gff, we used the potential gene models from *EVidenceModeler* and cross referenced these with several protein databases to validate and provide some curation of our gene models using *InterProScan* v 5.52–86.0 [95]. We used the following protein data bases: PFam, Panther, Prodom, Prosite, Tigrfams, Smart, Pirsf, Prints, Superfamily and CDD. Then searched the *EVidenceModeler* results using blastn/blastp against the blast-nt database, SwissProt, TrEMBL, orthodb10_arthropoda, the results of which we only keep if one or more has a hit with e-val > 1e-6 and then also match a protein domain from *InterProScan*. The best alignments from each database were used to create the final gene annotation result.

### Synteny across coleopteran and Insecta chromosome-level genomes

To examine the gene synteny between other Coleoptera genomes, we downloaded chromosome-level genomes from NCBI or supplied form the journal or authors website (see Table A in S1 Insecta Trees and Calibrations) [7–10,96]. We also used the unpublished genome assemblies (*Tribolium castaneum* [GCF_000002335.3], *Bombyx mori* [GCA_000151625.1], *Clogmia albipunctata* [clogmia.6], *Culex quinquefasciatus* [CpipJ3], and *Rhodnius prolixus* [Rhodnius_prolixus-3.0.3] as well as several others see Table A in S1 Insecta Trees and Calibrations, generated by the DNA Zoo Consortium (dnazoo.org). The assemblies were based on the whole genome sequencing data from [10,97–100] as well as Hi-C data generated by the DNA Zoo Consortium and assembled using 3D-DNA [14] and Juicebox Assembly Tools [21]. Next, we identified the BUSCO v.2 loci, (1658 Insecta gene set) and extracted their coordinates for the single and fragmented loci. We then compared the coordinates of *Pachyrhynchus sulphureomaculatus* to the other Coleoptera genomes. Following, we calculated the number of loci found in *P*. *sulphureomaculatus* chromosomes and those in the other Coleoptera and calculated the percent conserved within a chromosome. To visualize the shared synteny, we plotted the different pairs using the R package *RIdeogram* [101].

Next, we investigated whether the observed synteny was distinctive within Coleoptera relative to other orders of insects, such as Lepidoptera, in which high levels of synteny between taxa have been recorded [23,28]. We used all insect genomes (with some exceptions) available from NCBI that were marked as “chromosome” level. (See Table A in S1 Insecta Trees and Calibration for a complete list.) We tried to sample evenly across insect orders. For example, we excluded the many *Drosophila* genomes as they are all phylogenetically close relatives, and this would cause over-representation (i.e., we want patterns of chromosomal evolution across Diptera, not just *Drosophila*). Instead, we sampled individual species across the phylogenetic breadth of the genus. In addition, we also gathered genomes from the literature. (See Table A in S1 Insecta Trees and Calibration) Next, we identified all BUSCO version 5-beta loci that were single copy and calculated the gene order conservation (GOC) score (see https://m.ensembl.org/) using a custom script (see Scripts A and B in S1 Scripts). We then only consider BUSCO genes localized in chromosomes. First, we ordered the BUSCO v5-beta genes by scaffold and position and then identified two genes upstream and downstream from a particular gene. Next, to determine if a set of 4 genes are in the same order in our target genome, they receive a score of 1, 0.75, 0.5, 0.25 or 0 based on whether 4, 3, 2, 1 or 0 genes are in the same order, respectively. Missing genes between the two genomes are discarded from comparisons. This process is repeated along the length of the two genomes. We then summed the scores for the four categories 0–100% and added these categories together (e.g., if 8 matched *sets* were found at 25% and 1 at 100%, the total score would be 5). We computed the total GOC scores for all pairwise comparisons among the 143 taxa. Next, to consider the effect of the phylogenetic relationships, we reconstructed the relationship among our taxa using the BUSCO gene sets’ amino acids. We used custom scripts to identify a 50% complete matrix and used *mafft* with 1000 iterations and the “localpair” settings to align the sequences. Next, we used *trimAI* [102] with “automated1” settings to remove ambiguously aligned positions. RAxML-ng [103] with the LG+G8+F site rate substitution model was used to reconstruct the phylogeny for our exemplar taxa across Insecta. We dated the tree using dates (95% highest posterior density interval HPD) from previous studies [5,45,104] using the R package *ape* v.5.4 ‘makeChronosCalib*’* function [105] (see Tree A and Table B in S1 Insecta Trees and Calibrations for dates). Calibration points can be found in Table B in S1 Insecta Trees and Calibrations, from [5,45,104]. This calibration was done for visualization purposes only for the Coleoptera clade, as subsequent analyses do not use an ultrametric tree.

### Synteny decay rate analysis

#### Regression model methods

We would like to know how synteny decays with phylogenetic distance and if different orders show different patterns of decay. To accomplish this, we will evaluate whether the decay in synteny is best fit by a linear, exponential, or power law relationship with phylogenetic distance using least squares regression models. However, because the pairwise distances (both along phylogenetic branches and in genomic position of genes) violate the independence assumptions of ordinary least squares regression models, we will use a permutational approach to evaluate the significance of the regression models we fit. This approach is consistent with widespread methods in ecology and evolutionary biology that perform regression analyses with distance matrices [46,47].

#### Permutational algorithm

We implement this permutational approach using a custom algorithm in the R programming language [106]. We use a custom algorithm because our analytical set-up is slightly different from other approaches, e.g., [46,47,107]. Unlike existing approaches, we are not making all pairwise comparisons, but rather only comparisons within orders (not across orders); we are also interested in the effect of one distance matrix (phylogeny) on another distance matrix (synteny) in combination with a categorical factor (taxonomic order).

We are forced to take a permutational approach because synteny can only be quantified in a pairwise fashion, obviating other methods such as independent contrasts (Harmon & Glor 2010). We use a simple permutation algorithm that does not take into account phylogenetic branch lengths [108] (unlike e.g., Harmon & Glor 2010 [107]) because phylogenetic distance is a key explanatory variable and constraining it in the permutations would lead to nonsensical null distributions. Our permutational algorithm leaves the structure of the phylogeny taxonomic classifications unaltered while permuting levels of divergence in synteny across the tips.

We evaluate which model (linear, exponential, or power law) best fits the data using a permutational estimate of the F statistic (i.e. the ratio of variance explained by the model versus residual variance) and its deviation from the null. We use the F statistic instead of AIC or BIC because these information theoretic and Bayesian model comparison criteria have been shown to perform poorly in distance matrix regression settings [109]. Similarly, to evaluate whether insect orders have different rates of decay in synteny we again use permutational tests based on F statistics (full R code and data found in Doc A in S1 Synteny Analyses). Code was first validated by comparing our calculations to standard R functions using simulated data. After validating code (F -statistics in agreement), we then analyzed how synteny decays with phylogenetic distance, and whether different orders behave differently. Because Lepidoptera represent the majority of data (n = 3,081 out of 3,600 total data points), we also analyze the relationship between synteny and phylogenetic distance in the subset of data excluding Lepidoptera. We proceeded only with the exponential model as this proved to be the best fitting model.

## Supporting information

S1 Anno Results Contains the results of the genome annotation. The faa, gff, and model scores results files as well as trna sequences of *P*. *sulphureomaculatus* assembly. A: Table_A.gff: the gff file. B: Table_B.faa: the faa file. C: Table_C.tsv: the gene model scores file. D: Table_D.trna: the trna seqs.
(ZIP)

S1 BUSCO Analyses Results Contains: Table_A.xlsx, Table_B.csv. A: Table_A.xlsx: lists the BUSCO results from the different transcriptome assemblies by method used. B: Table_B.csv: lists the BUSCO results for the different versions of BUSCO insect e.g. 2, V4 and the associate percentages for single copy complete, complete and duplicated, fragmented and missing genes.
(ZIP)

S1 Fig *P*. *sulphureomaculatus* scaffold bubble plot of coverage versus GC content. Scaffolds included are from the unfiltered assembly. Taxonomic annotation provided via blastn alignment to the NCBI nt database.
(PDF)

S2 Fig Stacked bar plot of Insecta BUSCO gene sets by category for chromosome-level beetle genomes. Y-axis is the percent of BUSCO genes, X-axis labels are the genus names. The abbreviations in the legend are: D = duplicated, F = fragmented, M = missing and S = single.
(PDF)

S1 Insecta Trees and Calibrations Contains. A: Tree_A.newick: chronogram used in Fig 4. B: Table_A.xlsx: list of taxa used in synteny analyses by order, genus and species. With associate NCBI reference or similar. C: Table_B.xlsx: calibration points used to create “Tree_A.newick”, tree. D: Tree_B.tre: all Insecta tree used in Fig 7 and synteny analyses.
(ZIP)

S1 P sulph HiC heatmap all chroms & scaffolds Hi-C contact heat map of full assembly for *P*. *sulphureomaculatus*. (PDF)

S1 P79 coI.fasta Sequenced used to as seed DNA to extract mtDNA from assembly. (FASTA)

S1 Raw Data Reports Contains: Table_A.xlsx, Table_B.docx. A: Table_A.xlsx: Raw data report for PacBio sequences. B: Table_B.docx: Summary of Hi-C reads mapped.
(ZIP)

S1 RepeatMasker Results Contains the RepeatMasker result tables: Table_A.xlsx, Table_B.docx. A: Table_A.xlsx: The NCBI accession numbers used in repeatmasker analyses. B: Table_B.docx: Table of results from RepeatMasker for *P*. *sulphureomaculatus*.
(ZIP)

S1 Scripts Contains: Script_A.sh, Script_B.sh. A: Script_A.sh: script to create scaffold ordered BUSCOs. B: Script_B.sh: uses results from Script_B.sh to compute synteny scores.
(ZIP)

S1 Synteny Analyses Contains: A: Table_A.txt: the GOC pairwise distances matrix. B: Doc_A.pdf: instruction on how to preform synteny analyses. C: “synteny analyses/synteny/data/Insecta_matrix_matched_to_phylo_mod3.txt”: GOC pairwise distances matrix. D: “synteny analyses/synteny/data/rescaled_tree_insecta6.csv”: pairwise phylogenetic distance matrix. E: “synteny analyses/synteny/R/syntPermAOV”: R function to perform correlation of GOC distance and phylogenetic distance by insect order. F: “Read_me_Example_by A. Rominger synteny_perm.pdf” step by step instruction on how synteny correlations were performed.
(ZIP)
