# Supplementary material for: The Easter Egg Weevil (Pachyrhynchus) genome reveals syntenic patterns in Coleoptera across 200 million years of evolution
Source: PLoS Genet. 2021 Aug 30;17(8):e1009745. doi: 10.1371/journal.pgen.1009745 (PMC8432895; doi:10.1371/journal.pgen.1009745)
Supplement: S1 Fig — Scaffolds included are from the unfiltered assembly. Taxonomic annotation provided via blastn alignment to the NCBI nt database. (PDF) [file pgen.1009745.s003.pdf]

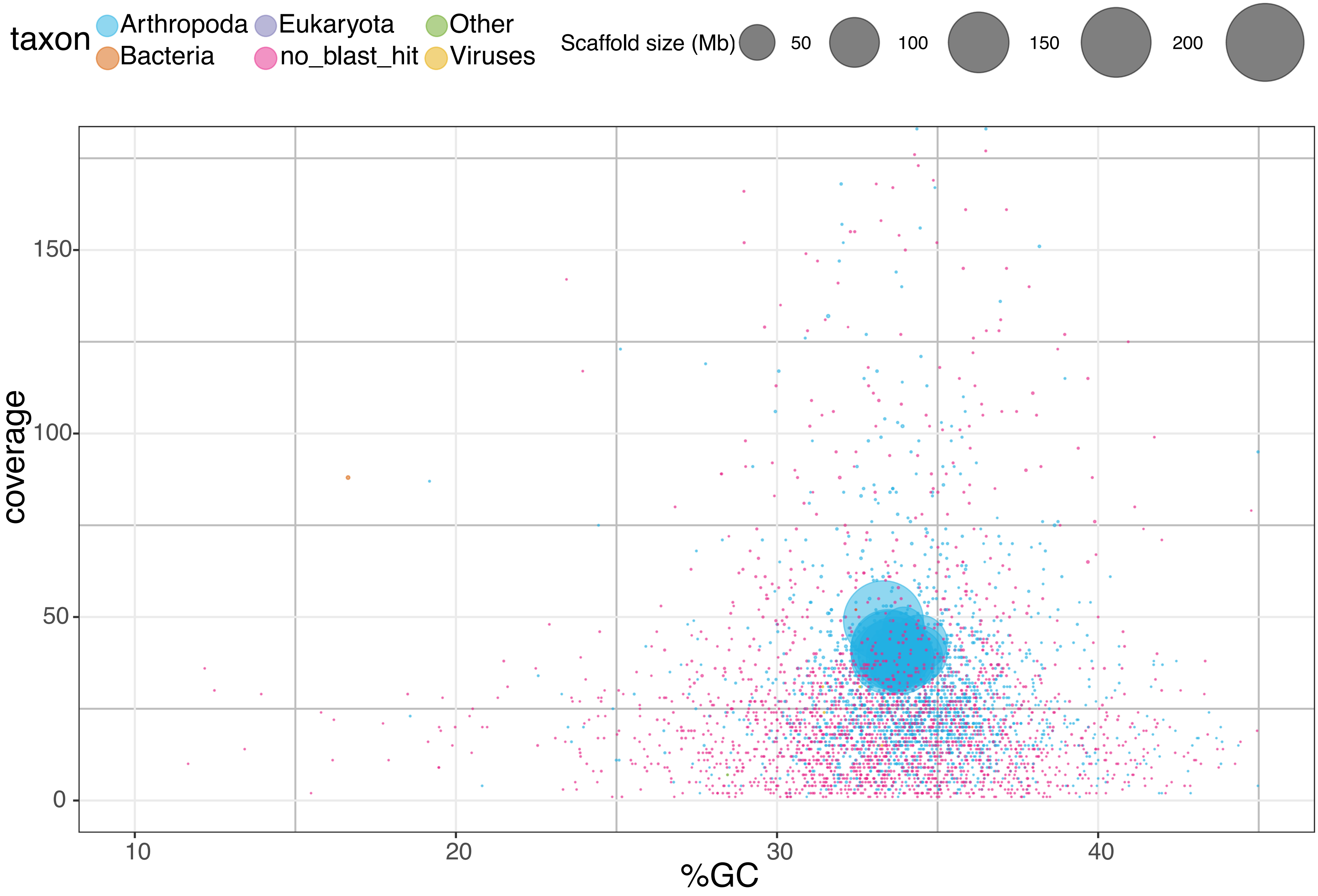

Figure S1. P. sulphureomaculatus scaffold bubble plot of coverage versus GC content. Scaffolds included are from the unfiltered assembly. Taxonomic annotation provided via blastn alignment to the NCBI nt database.
