# Supplementary material for: The Easter Egg Weevil (Pachyrhynchus) genome reveals syntenic patterns in Coleoptera across 200 million years of evolution
Source: PLoS Genet. 2021 Aug 30;17(8):e1009745. doi: 10.1371/journal.pgen.1009745 (PMC8432895; doi:10.1371/journal.pgen.1009745)
Supplement: S2 Fig — Y-axis is the percent of BUSCO genes, X-axis labels are the genus names. The abbreviations in the legend are: D = duplicated, F = fragmented, M = missing and S = single. (PDF) [file pgen.1009745.s004.pdf]

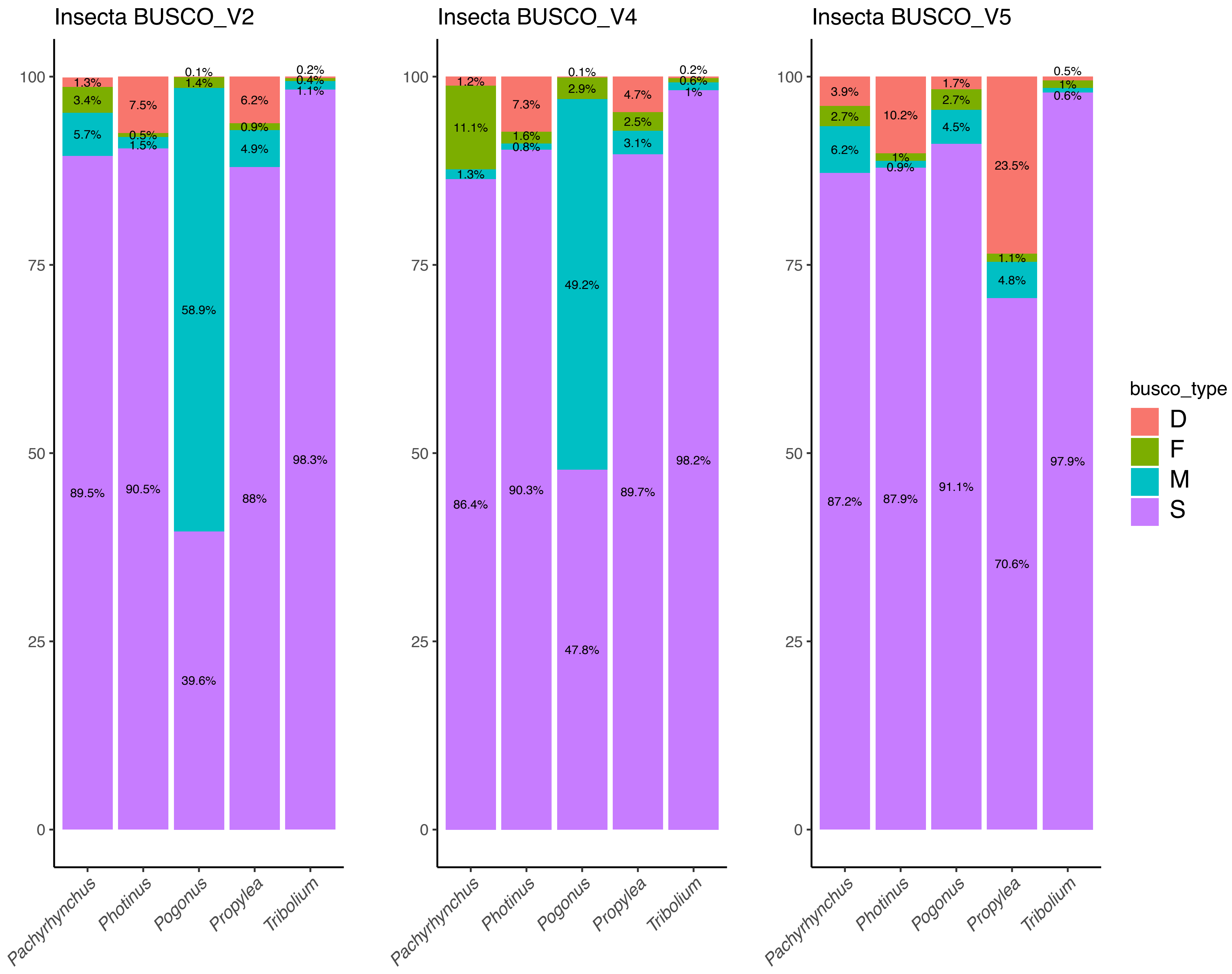

Figure S2. Stacked bar plot of Insecta BUSCO gene sets by category for chromosome-level beetle genomes. Y-axis is the percent of BUSCO genes, X-axis labels are the genus names. The abbreviations in the legend are: D=duplicated, F=fragmented, M=missing and S=single
