# Supplementary figures and images for: The Easter Egg Weevil (Pachyrhynchus) genome reveals syntenic patterns in Coleoptera across 200 million years of evolution
Source: PLoS Genet. 2021 Aug 30;17(8):e1009745. doi: 10.1371/journal.pgen.1009745 (PMC8432895; doi:10.1371/journal.pgen.1009745)

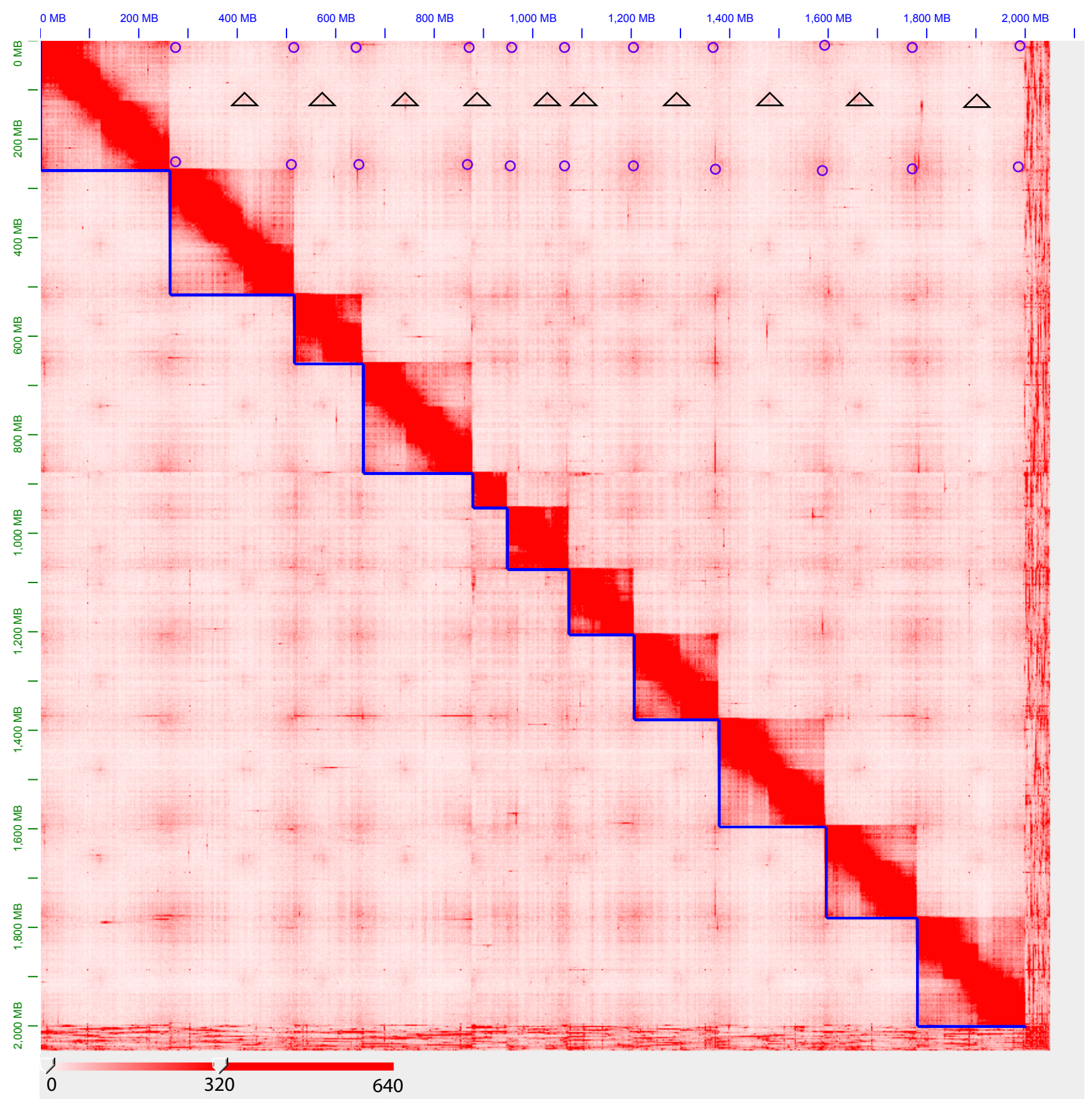

Supplement: S1 P sulph HiC heatmap all chroms & scaffolds — (PDF) [file pgen.1009745.s006.pdf]
