# Supplementary material for: The Easter Egg Weevil (Pachyrhynchus) genome reveals syntenic patterns in Coleoptera across 200 million years of evolution
Source: PLoS Genet. 2021 Aug 30;17(8):e1009745. doi: 10.1371/journal.pgen.1009745 (PMC8432895; doi:10.1371/journal.pgen.1009745)
Supplement: S1 Raw Data Reports — Contains: Table_A.xlsx, Table_B.docx. A: Table_A.xlsx: Raw data report for PacBio sequences. B: Table_B.docx: Summary of Hi-C reads mapped. (ZIP) [file pgen.1009745.s008.zip › S1_Raw_Data_Reports/Table_B.docx]

| Inter-chromosomal: 56,711,177 (24.85% / 38.63%) |
| --- |
| Intra-chromosomal: 23,941,704 (10.49% / 16.31%) |
| Short Range (<20Kb): 18,227,329 (7.99% / 12.42%) |
| Long Range (>20Kb): 5,714,353 (2.50% / 3.89%) |

**Supplementary Table 1A**. Summary of Hi-C reads mapped.
