# Supplementary material for: The Easter Egg Weevil (Pachyrhynchus) genome reveals syntenic patterns in Coleoptera across 200 million years of evolution
Source: PLoS Genet. 2021 Aug 30;17(8):e1009745. doi: 10.1371/journal.pgen.1009745 (PMC8432895; doi:10.1371/journal.pgen.1009745)
Supplement: S1 RepeatMasker Results — Contains the RepeatMasker result tables: Table_A.xlsx, Table_B.docx. A: Table_A.xlsx: The NCBI accession numbers used in repeatmasker analyses. B: Table_B.docx: Table of results from RepeatMasker for P. sulphureomaculatus. (ZIP) [file pgen.1009745.s009.zip › S1_RepeatMasker_Results/Table_B.docx]

RepeatMasker results

number of length percentage

elements* occupied of sequence

SINEs: 14405 2032333 bp 0.10 %

ALUs 2 100 bp 0.00 %

MIRs 5 220 bp 0.00 %

LINEs: 904430 426128030 bp 20.77 %

LINE1 18686 3751928 bp 0.18 %

LINE2 82585 26106034 bp 1.27 %

L3/CR1 33062 19638536 bp 0.96 %

LTR elements: 146818 54238591 bp 2.64 %

ERVL 939 40708 bp 0.00 %

ERVL-MaLRs 5 242 bp 0.00 %

ERV_classI 22143 1491030 bp 0.07 %

ERV_classII 3018 178304 bp 0.01 %

DNA elements: 2193709 676562170 bp 32.97 %

hAT-Charlie 58197 33317516 bp 1.62 %

TcMar-Tigger 30866 17743614 bp 0.86 %

Unclassified: 1402764 341139600 bp 16.63 %

Total interspersed repeats:1500100724 bp 73.11 %

Small RNA: 5277 408769 bp 0.02 %

Satellites: 6319 1369641 bp 0.07 %

Simple repeats: 277 29490 bp 0.00 %

Low complexity: 0 0 bp 0.00 %
