# Supplementary material for: The Easter Egg Weevil (Pachyrhynchus) genome reveals syntenic patterns in Coleoptera across 200 million years of evolution
Source: PLoS Genet. 2021 Aug 30;17(8):e1009745. doi: 10.1371/journal.pgen.1009745 (PMC8432895; doi:10.1371/journal.pgen.1009745)
Supplement: S1 Synteny Analyses — Contains: A: Table_A.txt: the GOC pairwise distances matrix. B: Doc_A.pdf: instruction on how to preform synteny analyses. C: “synteny analyses/synteny/data/Insecta_matrix_matched_to_phylo_mod3.txt”: GOC pairwise distances matrix. D: “synteny analyses/synteny/data/rescaled_tree_insecta6.csv”: pairwise phylogenetic distance matrix. E: “synteny analyses/synteny/R/syntPermAOV”: R function to perform correlation of GOC distance and phylogenetic distance by insect order. F: “Read_me_Example_by A. Rominger synteny_perm.pdf” step by step instruction on how synteny correlations were performed. (ZIP) [file pgen.1009745.s011.zip › S1_Synteny_Analyses/synteny/Read_me_Example_by A. Rominger synteny_perm.pdf]

Synteny decay rate analysis

Regression model methods

We would like to know how synteny decays with phylogenetic distance and if different orders show different patterns of decay. We will evaluate whether the decay in synteny is best fit by a linear, exponential, or power law relationship with phylogenetic distance using least squares regression models. However, because the pairwise distances (both along phylogenetic branches and in genomic position of genes) violate the independence assumptions of ordinary least squares regression models, we will use a permutational approach to evaluate the significance of the regression models we fit. This approach is consistent with widespread methods in ecology and evolution that preform regression analyses with distance matrices (Lichstein 2007; McArtor *et al.* 2017).

Permutational algorithm

We implement this permutational approach using a custom algorithm in the R programming language (R Core Team 2020). We use a custom algorithm because our analytical set-up is slightly different than other approaches (e.g., Lichstein 2007; Harmon & Glor 2010; McArtor *et al.* 2017). Unlike existing approaches we are not making all pairwise comparisons, but rather only comparisons within orders (not across orders); we are also interested in the effect of one distance matrix (phylogeny) on another distance matrix (synteny) in combination with a categorical factor (taxonomic order).

We are forced to take a permutational approach because synteny can only be quantified in a pairwise fashion, obviating other methods such as independent contrasts (Harmon & Glor 2010). We use a simple permutation algorithm that does not take into account phylogenetic branch lengths (Lapointe & Garland 2001; unlike e.g., Harmon & Glor 2010) because phylogenetic distance is a key explanatory variable and constraining it in the permutations would lead to nonsensical null distributions. Our permutational algorithm leaves the structure of the phylogeny taxonomic classifications unaltered while permuting levels of divergence in synteny across the tips.

We evaluate which model (linear, exponential, or power law) best fits the data using a permutational estimate of the F statistic (i.e. the ratio of variance explained by the model versus residual variance) and its deviation from the null. We use the F statistic instead of AIC or BIC because these information theoretic and Bayesian model comparison criteria have been shown to preform poorly in distance matrix regression settings (Franckowiak *et al.* 2017). Similarly, to evaluate whether orders have different rates of decay in synteny we again use permutational tests based on F statistics. The code implementing our permutational algorithm is reproduced below.

```r
#' Permutational ANOVA for specific analysis of relationship between
#' synteny and phylogenetic distance across different order
#'
#' @param treeD vector of phylogenetic distances
#' @param syntD vector of synteny distances
#' @param ord vector of order names
#' @param B number of permutations to preform
#' @return returns a data.frame similar to a standard ANOVA table

syntPermAOV <- function(treeD, syntD, ord, B = 999) {
    dat <- data.frame(y = syntD, x = treeD, g = factor(ord))
    nord <- nlevels(dat$g)
```

```r
    df <- c(1, rep(nord - 1, 2))
    df <- c(df, nrow(dat) - sum(df) - 1)

    # make models of reducing complexity from full (X3) to slope only (X1)
    X3 <- model.matrix(y ~ x * g, data = dat)
    X2 <- X3[, 1:(2 + nord - 1)]
    X1 <- X3[, 1:2]

    # do matrix calculations on different models
    pHat1 <- partialHat(X1)
    pHat2 <- partialHat(X2)
    pHat3 <- partialHat(X3)

    # calculate F statistics
    fobs <- fval(list(pHat1, pHat2, pHat3),
                 list(X1, X2, X3),
                 df, dat$y)

    # calculate null F distributions
    fnull <- lapply(1:B, function(i) {
        newy <- sample(dat$y)
        f <- fval(list(pHat1, pHat2, pHat3),
                  list(X1, X2, X3),
                  df, newy)
        return(f)
    })

    fnull <- do.call(rbind, c(fnull, fobs))

    # calculate pvals from fnull
    pval <- numeric(length(fobs))
    for(i in 1:length(pval)) {
        pval[i] <- mean(fnull[, i] >= fobs[i])
    }

    tab <- data.frame(DF = paste(c(df[1:3], sum(df[1:3])), df[4], sep = ', '),
                      FVal = fobs, Pval = pval)

    return(tab)
}


#' preforms part of the matrix calculation needed to solve for the parameters of
#' a linear regression; the full solution is
#' `bHat <- solve(t(X) %*% X) %*% t(X) %*% y`. We leave out the part about `y`
#' because we are going to permute `y`, but all calculations not involving `y`
#' can be done once to save computation
#' @param X the model matrix
#' @note only intended for use inside `syntPermAOV`

partialHat <- function(X) {
    return(solve(t(X) %*% X) %*% t(X))
}
```

```r
#' calculate predicted y using the output of `partialHat`
#' @param pHat the output of `partialHat`
#' @param X the model matrix
#' @param y the observed (possibly permuted) response variable
#' @note only intended for use inside `syntPermAOV`

yhat <- function(pHat, X, y) {
    b <- pHat %*% y
    return(X %*% b)
}


#' function to calculate F values
#' @param pHat the output of `partialHat`
#' @param X the model matrix
#' @param df vector of degrees of freedom
#' @param y the observed (possibly permuted) response variable
#' @note only intended for use inside `syntPermAOV`

fval <- function(pHat, X, df, y) {
    y1 <- yhat(pHat[[1]], X[[1]], y)
    y2 <- yhat(pHat[[2]], X[[2]], y)
    y3 <- yhat(pHat[[3]], X[[3]], y)

    ssq <- c(sum((y1 - mean(y))^2),
             sum((y2 - y1)^2),
             sum((y3 - y2)^2),
             sum((y3 - y)^2))

    msq <- ssq / df

    msqTot <- sum(ssq[1:3]) / sum(df[1:3])

    f <- c(msq[1:3], msqTot) / msq[4]

    return(f)
}
```

We validate our code by comparing our calculations to standard R functions using simulated data, reproduced below.

```r
x <- runif(500)
g <- rep(1:4, 25)
y <- g^3 + 5 * g * x + rnorm(100, sd = 2)

m <- lm(y ~ x * g, data = data.frame(y = y, x = x, g = factor(g)))

syntPermAOV(x, y, g, B = 999)
```

```
##        DF        FVal        Pval
## 1 1, 492  1687.18364 0.002991027
## 2 3, 492 29049.82224 0.000997009
## 3 3, 492    98.52169 0.003988036
```

```
## 4 7, 492 12733.17363 0.001994018
```

```r
anova(m)
```

```
## Analysis of Variance Table
##
## Response: y
##            Df Sum Sq Mean Sq   F value    Pr(>F)
## x           1   7073    7073  1687.184 < 2.2e-16 ***
## g           3 365356  121785 29049.822 < 2.2e-16 ***
## x:g         3   1239     413    98.522 < 2.2e-16 ***
## Residuals 492   2063       4
## ---
## Signif. codes:  0 '***' 0.001 '**' 0.01 '*' 0.05 '.' 0.1 ' ' 1
```

```r
summary(m)[['fstatistic']]
```

```
##    value    numdf    dendf
## 12733.17     7.00   492.00
```

The degrees of freedom and F-statistics all agree. The p-values differ in magnitude (but not in significance) but this discrepancy is to be expected when using finite permutations.

Analysis of synteny across phylogenetic distance and orders

We can now analyze how synteny decays with phylogenetic distance, and whether different orders behave differently. The code to run this analysis is reproduced below.

```r
# phylogenetic distances
treeD <- read.csv("data/rescaled_tree_insecta6.csv",
                  header = TRUE, row.names = 1)

# synteny distances
syntD <- read.table("data/Insecta_matrix_matched_to_phylo_mod3.txt",
                   header = TRUE, row.names = 1)

# map of location of orders in distance matrices
taxaii <- data.frame(ord = c('Hemiptera', 'Hymenoptera', 'Coleoptera',
                             'Diptera', 'Lepidoptera'),
                     i0 = c(2, 16, 42, 49, 65),
                     i1 = c(14, 40, 48, 64, 143))

# function to extract lower triangles for a given order
getLowerTri <- function(ord) {
    o <- which(taxaii$ord == ord)
    ii <- taxaii$i0[o]:taxaii$i1[o]

    mt <- treeD[ii, ii]
    ms <- syntD[ii, ii]

    return(data.frame(ord = ord,
                      treeD = mt[lower.tri(mt)],
                      syntD = ms[lower.tri(ms)]))
}


# make data.frame with all data on distances and groups
```

```r
ordDat <- lapply(taxaii$ord, getLowerTri)
ordDat <- do.call(rbind, ordDat)


# make three different models: linear, exponential, power law

# linear
yLin <- ordDat$syntD
xLin <- ordDat$treeD
ord <- ordDat$ord
modLin <- syntPermAOV(xLin, yLin, ord, B = 9999)

# exponential
yExp <- log(ordDat$syntD)
modExp <- syntPermAOV(xLin, yExp, ord, B = 9999)

# power law
xExp <- log(ordDat$treeD)
modPow <- syntPermAOV(xExp, yExp, ord, B = 9999)
```

Because Lepidoptera represent the majority of data ($n = 3{,}081$ out of 3,600 total data points), we also analyze the relationship between synteny and phylogenetic distance in the subset of data excluding Lepidoptera. We do this only with the exponential model as this proved to be the best fitting model (see results section). The code to reproduce this analysis without Lepidoptera is below.

```r
modExpNoLep <- syntPermAOV(xLin[ord != 'Lepidoptera'],
                           yExp[ord != 'Lepidoptera'],
                           ord[ord != 'Lepidoptera'],
                           B = 9999)
```

Regression model results

The exponential decay model has the highest total model F-statistic and smallest p-value $F_{9,3590} = 15{,}111$, $p = 2 \times 10^{-4}$ (compared to linear: $F_{9,3590} = 3{,}493$, $p = 3 \times 10^{-4}$; power law: $F_{9,3590} = 12{,}165$, $p = 3 \times 10^{-4}$). This supports the exponential model as the best fitting model for the relationship between synteny and phylogenetic distance.

Using this best fitting exponential model we can now ask whether different orders show different rates of decay, again using permutational F-statistics. We find that the interaction between phylogenetic distance and order identity is statistically significant: $F_{4,3590} = 1{,}344$, $p = 4 \times 10^{-4}$. We also find that this result is not driven solely by Lepidoptera; the analysis excluding Lepidoptera still finds a significant interaction between phylogenetic distance and order: $F_{3,511} = 39$, $p = 4 \times 10^{-4}$.

Finally, we can make a plot of the data and the best fitting exponential model, shown in Figure 1 with code reproduced below. Note that because the best model is exponential we plot the data with a log-transformed y-axis.

```r
# load a custom library with a function for making pretty log axes
s <- require(socorro)
if(!s) {
    devtools::install_github('ajrominger/socorro')
    library(socorro)
}

# the model fit by `syntPermAOV` has the same parameter values as given by `lm`
# so we can use the functionality of `lm` to plot predictions
```

```r
mod <- lm(y ~ x * g, data = data.frame(x = xLin, y = yExp, g = ord))

# make uniform predictor variables for prediction
uniqueOrd <- sort(unique(ord))
xnew <- lapply(uniqueOrd, function(o) {
    xr <- range(xLin[ord == o])
    out <- data.frame(x = seq(xr[1], xr[2], length.out = 50),
                      g = o)

    return(out)
})

xnew <- do.call(rbind, xnew)


# custom function to draw predicted curves
syntCurve <- function(ord, ...) {
    thisx <- xnew[xnew$g == ord, ]
    thisy <- exp(predict(mod, thisx))

    lines(thisx$x, thisy, col = factor(ord, levels = uniqueOrd), ...)
}

# a nice color palette
pal <- list(h = c(1, 0.02, 0.78, 0.6, 0.5),
            s = c(0, 0.70, 1.00, 1, 1),
            v = c(0, 0.80, 0.90, 1, 1))

padj <- 0.8
palette(hsv(pal$h, pal$s * padj, pal$v))

# the plot
par(mar = c(3, 3, 0, 0) + 0.25, mgp = c(2, 0.5, 0), tcl = -0.25)

plot(xLin, yLin, col = factor(ord), lwd = 1.5,
     xlab = 'Rescaled phylogenetic distance',
     ylab = 'Rescaled synteny distance',
     log = 'y', yaxt = 'n',
     ylim = c(10, 10^4))

logAxis(2, expLab = TRUE)

# add legend
legend('topright', legend = uniqueOrd, x.intersp = 1.5,
       col = factor(uniqueOrd), pch = 1, pt.lwd = 1.5, pt.cex = 1.2,
       bty = 'n')

# add lines
ladj <- 0.8
palette(hsv(pal$h, pal$s, pal$v * ladj))
for(o in uniqueOrd) {
    syntCurve(o, lwd = 3)
}
```

```r
# add lines to legend
legend('topright', legend = uniqueOrd, text.col = 'transparent',
       col = factor(uniqueOrd), lty = 1, lwd = 3, seg.len = 1,
       bty = 'n')
```

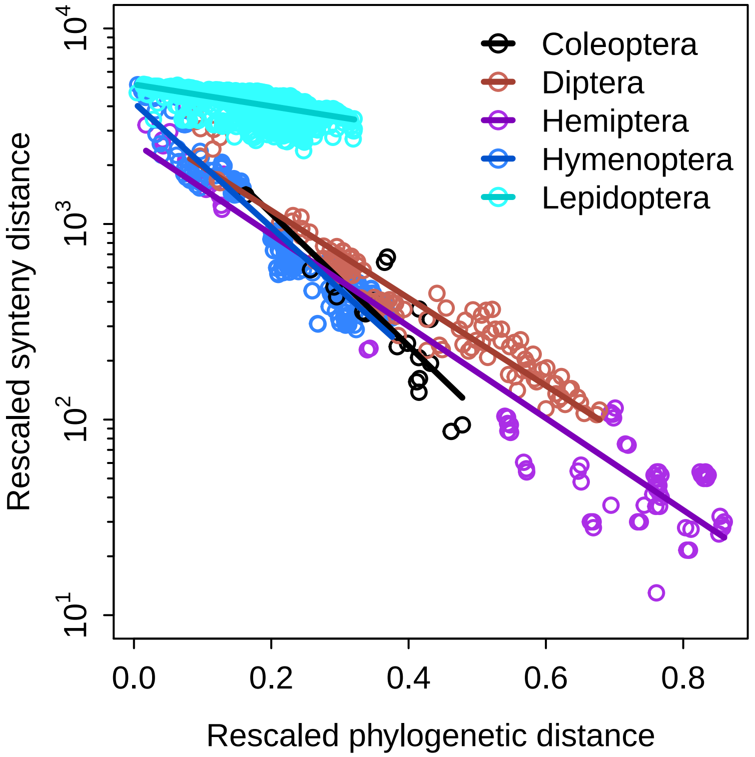

Figure 1: Relationship between synteny and phylogenetic distance across different insect orders. Lines show the best-fitting exponential decay model. Note the log-transformed y-axis.

References

Franckowiak RP, Panasci M, Jarvis KJ, *et al.* (2017) Model selection with multiple regression on distance matrices leads to incorrect inferences. *PLoS One*, **12**, e0175194.

Harmon LJ, Glor RE (2010) Poor statistical performance of the mantel test in phylogenetic comparative analyses. *Evolution: International Journal of Organic Evolution*, **64**, 2173–2178.

Lapointe F-J, Garland T (2001) A generalized permutation model for the analysis of cross-species data. *Journal of Classification*, **18**, 109–127.

Lichstein JW (2007) Multiple regression on distance matrices: A multivariate spatial analysis tool. *Plant Ecology*, **188**, 117–131.

McArtor DB, Lubke GH, Bergeman C (2017) Extending multivariate distance matrix regression with an effect size measure and the asymptotic null distribution of the test statistic. *Psychometrika*, **82**, 1052–1077.

R Core Team (2020) *R: A language and environment for statistical computing.* R Foundation for Statistical Computing, Vienna, Austria.
